# Artificial intelligence in fracture detection with different image modalities and data types: A systematic review and meta-analysis

**DOI:** 10.1371/journal.pdig.0000438

**Published:** 2024-01-30

**Authors:** Jongyun Jung, Jingyuan Dai, Bowen Liu, Qing Wu

**Affiliations:** 1 Department of Biomedical Informatics (Dr. Qing Wu, Jongyun Jung, and Jingyuan Dai), College of Medicine, The Ohio State University, Columbus, Ohio, United States of America; 2 Department of Mathematics and Statistics, Division of Computing, Analytics, and Mathematics, School of Science and Engineering (Bowen Liu), University of Missouri-Kansas City, Kansas City, Missouri, United States of America; University of Washington, UNITED STATES

## Abstract

Artificial Intelligence (AI), encompassing Machine Learning and Deep Learning, has increasingly been applied to fracture detection using diverse imaging modalities and data types. This systematic review and meta-analysis aimed to assess the efficacy of AI in detecting fractures through various imaging modalities and data types (image, tabular, or both) and to synthesize the existing evidence related to AI-based fracture detection. Peer-reviewed studies developing and validating AI for fracture detection were identified through searches in multiple electronic databases without time limitations. A hierarchical meta-analysis model was used to calculate pooled sensitivity and specificity. A diagnostic accuracy quality assessment was performed to evaluate bias and applicability. Of the 66 eligible studies, 54 identified fractures using imaging-related data, nine using tabular data, and three using both. Vertebral fractures were the most common outcome (n = 20), followed by hip fractures (n = 18). Hip fractures exhibited the highest pooled sensitivity (92%; 95% CI: 87–96, *p<* 0.01) and specificity (90%; 95% CI: 85–93, *p<* 0.01). Pooled sensitivity and specificity using image data (92%; 95% CI: 90–94, *p<* 0.01; and 91%; 95% CI: 88–93, *p <* 0.01) were higher than those using tabular data (81%; 95% CI: 77–85, *p<* 0.01; and 83%; 95% CI: 76–88, *p <* 0.01), respectively. Radiographs demonstrated the highest pooled sensitivity (94%; 95% CI: 90–96, *p <* 0.01) and specificity (92%; 95% CI: 89–94, *p<* 0.01). Patient selection and reference standards were major concerns in assessing diagnostic accuracy for bias and applicability. AI displays high diagnostic accuracy for various fracture outcomes, indicating potential utility in healthcare systems for fracture diagnosis. However, enhanced transparency in reporting and adherence to standardized guidelines are necessary to improve the clinical applicability of AI.

**Review Registration**: PROSPERO (CRD42021240359).

## Author summary

Artificial Intelligence (AI) is increasingly employed to detect fractures by using various imaging modalities and data types. Our search of Medline (via PubMed), Web of Science, and IEEE revealed numerous primary studies demonstrating AI’s superior performance in fracture detection. This systematic review and meta-analysis is the first to assess and compare the diagnostic accuracy of AI models across different imaging modalities and data types for various fracture outcomes. We found that AI models achieve high accuracy in fracture detection, particularly with radiograph images. However, we identified significant flaws in study design and reporting, limiting real-world applicability. Few studies provided patient characteristics, and only half reported the hyperparameter selection process. Our findings underscore the benefits of using AI models with radiographs for fracture detection, as they outperform other imaging modalities. Despite similar results across modalities, inadequate methodology and reporting in AI model evaluations call for improvement. Considering AI’s high diagnostic performance, integrating it into existing fracture risk assessment tools could enhance patient identification and enable early intervention.

## Introduction

Bone fractures represent a significant public health concern globally [1], particularly for individuals with osteoporosis [2]. Fractures contribute to work absences, disability, reduced quality of life, health complications, and increased healthcare costs, affecting individuals, families, and societies [3,4]. A meta-analysis of 113 studies reported the pooled cost of hospital treatment for a hip fracture after 12 months as $10,075, with total health and social care costs amounting to $43,669 per hip fracture [5].

Artificial Intelligence (AI), encompassing Machine Learning (ML) and Deep Learning (DL), has been extensively employed for fracture outcome prediction due to technological advancements and accessibility. Various imaging modalities, including X-rays [6,7], computed tomography (CT) [8,9], and magnetic resonance imaging (MRI) [10,11], have been used in fracture diagnosis and detection. AI can also predict fractures using tabular data, such as electronic medical records (structured patient-level data). However, few studies [12–14] have applied AI with tabular data in fracture prediction despite its growing importance over the past decade. Recent systematic reviews and meta-analyses have reported high accuracy for AI in fracture detection and classification. Kuo et al. [15] summarized 42 studies with 115 contingency tables, finding pooled sensitivity of 92% (95% CI: 88, 94) and specificity of 91% (95% CI: 88, 93). Yang et al. [16] reviewed 14 studies on orthopedic fractures, reporting pooled sensitivity and specificity of DL models as 87% (95% CI: 78, 93) and 91% (95% CI: 85, 95), respectively.

However, existing systematic review and meta-analysis studies focused solely on image-based analyses, neglecting comprehensive examination of various imaging modalities and data types (image, tabular, or both). Despite the superior performance of AI for medical image analysis and using tabular data, a critical gap exists in the current literature concerning the optimal choice of image modalities and the choice between image, tabular, or combined data types. There is a lack of comprehensive guidance on the most effective selection of image modalities and data types for fracture diagnosis. This gap in knowledge underscores the need for systematic investigation to determine which image modality, and by extension, which data type, yields the highest diagnostic accuracy and clinical relevance in AL algorithms. Addressing this gap will not only optimize the design of AI-based diagnostic tools but also enable healthcare practitioners to make informed decisions when selecting appropriate imaging modalities and data types for improved patient care.

Thus, this study primarily aims to evaluate the diagnostic accuracy of AI in fracture detection using diverse imaging modalities and data types, reflecting AI’s growing role in healthcare. Additionally, we seek to synthesize current evidence on AI-based fracture detection, offering a concise overview and discerning the strengths and limitations of various data types, whether image, tabular, or combined.

## Materials and methods

### Identification and selection of studies

This systematic review, registered with PROSPERO (CRD42021240359), follows PRISMA guidelines (**S1 PRISMA Checklist**) [17]. We searched Medline (via PubMed), Web of Science, and IEEE. The last search was conducted on December 15, 2022, and we manually searched bibliographies, citations, and related articles of included studies. **S1 Text** lists each search term. Two independent reviewers (JJ and JD) assessed study eligibility, resolving disagreements through discussion or involving a third author (BL) if necessary.

Eligible studies predicted fracture outcomes using structured patient-level health data (electronic health records and cohort studies data) and image-related data (MRI, DXA, and X-ray). We excluded reviews, gray literature, non-human subject studies, studies without machine learning or deep learning models, fracture outcomes, AUC, accuracy, sensitivity, specificity, validation, and insufficient algorithm development details. We only considered studies published in English without time restrictions.

### Data extraction

All three categories of data were considered: image-related, tabular, and both. Image-type studies used MRI, DXA, CT, or X-ray; tabular-type studies used structured electronic health records data; image and tabular studies used both data types. Two investigators (JJ and JD) independently evaluated study eligibility, extracting relevant data for articles meeting inclusion criteria. A structured data collection form was used to capture general study characteristics, population, data preprocessing, clinical outcomes, analytical methods, and results. A third author (BL) resolved discrepancies if necessary. We constructed the contingency table (true positive, true negative, false positive, and false negative) based on the provided information of sensitivity, specificity, positive predictive value, and negative predictive value for each study (**S4 Table**). If the study reported multiple sensitivity and specificity, we used the highest sensitivity and specificity.

### Statistical analysis

Meta-analyses were performed using a random-effects model to calculate the pooled sensitivity and specificity based on logit transformation [18,19], using the Clopper-Pearson interval to calculate 95% confidence intervals for each study [20]. We used a unified hierarchical summary receiver operating characteristic curve (HSROC) to investigate the relationship between logit-transformed sensitivity and specificity. We calculated the diagnostic odds ratio and used inverse variance weighting for pooling with random effect models [21].

### Sensitivity analysis

The logit transformation does not consider the correlation between sensitivity, specificity, and threshold effects; another model is desired to capture this missing part. Barendregt et al. [22] recommend using the Freeman-Tukey double arcsine transformation instead of the logit transformation. Hence, we used the Freeman-Tukey double arcsine transformation as a sensitivity analysis [22] for a random-effects model.

### Subgroup analysis

Two subgroup analyses were conducted: 1) three data types (images, tabular, or images and tabular) and 2) different image modalities among image data used in AI. Statistical analysis was performed using R [23], with ‘meta’ [24] and ‘mada’ [25] packages. A *p*-value of < 0.05 was considered statistically significant.

### Publication bias

We utilized the contour-enhanced funnel plot [26] to illustrate the assessment of publication bias for each fracture outcome and data type used. Each data point in the contour-enhanced funnel plot represents an individual study, and the plot incorporates contour lines that delineate expected areas of symmetry in the absence of bias. The plot provides insights into potential publication bias, with asymmetry suggesting a deviation from expected publication patterns. We employed the trim-and-fill method to address publication bias [22] further. This statistical approach helps adjust for the potential missing studies due to publication bias by imputing hypothetical “filled” studies and recalculating the effect size accordingly.

### Risk of bias and applicability

Two reviewers (JJ and JD) independently evaluated the risk of bias in each study using Quality Assessment of Diagnostic Accuracy Studies (QUADAS-2) [27], assessing four domains: patient selection, index test, reference standard, and flow and timing. The risk of applicability was evaluated with the first three domains.

## Results

### Study selection and characteristics

**Fig 1:**
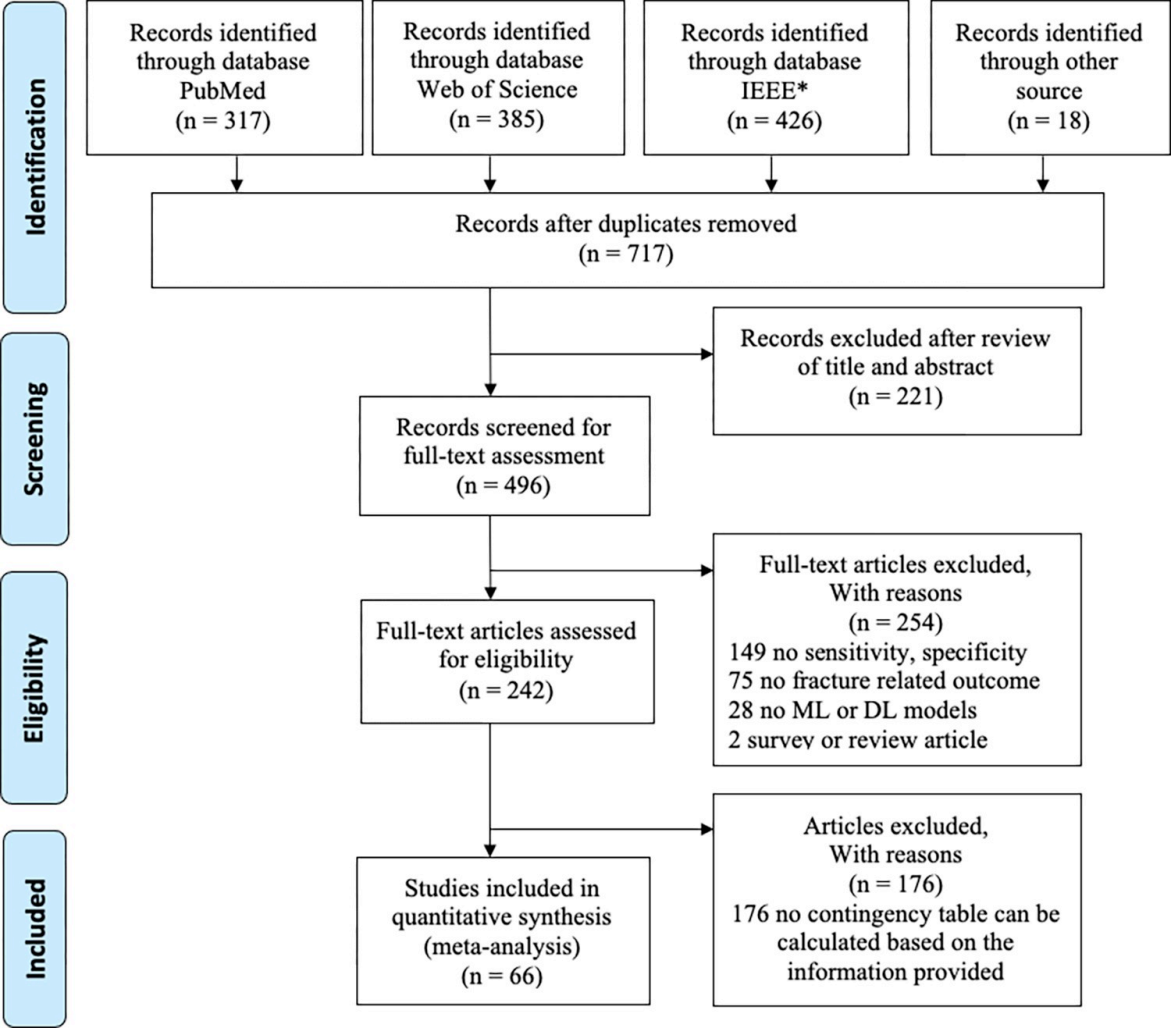
Flow chart of the literature selection in PubMed, Web of Science, and Institute of Electrical and Electronics Engineers (search conducted on December 15, 2022). *IEEE: Institute of Electrical and Electronics Engineers.

Our search identified 1,128 studies, yielding 717 unique ones after removing duplicates (**Fig 1**). We screened titles and abstracts and selected 496 studies for full-text review based on our inclusion criteria. We then excluded 254 studies for lacking sensitivity and specificity information (149 studies), not having fracture-related outcomes (75 studies), not using ML models (28 studies), or being survey or review articles (2 studies). We further removed 176 studies because no contingency table could be calculated from the provided information. Ultimately, 66 studies were included in our systematic review and meta-analysis.

The selected studies were published between 2007 and 2022, with 73% (48 studies) published in the last three years (**Table 1**). The studies were conducted in various countries, including Asian countries (26 studies) [6,9,11,28–50], North American countries (19 studies) [14,34,36,51–66], European countries (14 studies) [13,59,67–78], Australia (1 study) [79] and Brazil (2 studies) [10,80] (**Table 1**). Four studies did not provide the country information [81–84].

**Table 1:** Fracture detection of 66 selected studies using machine learning and deep learning models and general characteristics of the study.

First author (Year published)	Country	Data type	Outcome	Model
Almog et al. (2020) [12]	USA	Tabular	Osteoporotic Fracture	XGBoost, Ensemble
Bae et al. (2021) [7]	Canada	Image	Femoral Neck	CNN, Four Different Convolutional Block Attention Modules
Beyaz et al. (2020) [67]	Turkey	Image	Femoral Neck	CNN
Burns et al. (2017) [8]	USA	Image	Vertebral	SVM
Chen et al. (2021) [28]	Taiwan	Image	Vertebral	CNN
Chen et al. (2022) [46]	China	Image	Vertebral	CNN, Other: Used ResNetSt-50 as the backbone network of the baseline model
Cheng et al. (2019) [6]	Taiwan	Image	Hip	CNN
Cheng et al. (2020) [29]	Taiwan	Image	Hip	CNN
Cheng et al. (2021) [30]	Taiwan	Image	Hip	CNN
Choi et al. (2020) [47]	South Korea	Image	Supracondylar	CNN
Chou et al. (2022) [31]	Taiwan	Image	Vertebral	CNN, Transfer Learning, Ensemble model (ResNet34, DenseNet121, DenseNet201)
Chung et al. (2018) [45]	Korea	Image	Proximal humerus	CNN
Derkatch et al. (2019) [51]	Canada	Image	Vertebral	CNN
Galassi et al. (2020) [68]	Spain	Tabular+Image	Hip	LR, SVM, Decision Trees, Random Forest
Guermazi et al. (2022) [52]	USA	Image	Hip, Wrist, Pelvic, Thoracolumbar, Foot, Ankle, Arm, Shoulder, Rib	Detectron2
Gupta et al. (2020) [53]	USA	Image	Hip	Transfer Learning: used VGG16 architecture with pre-trained weights using the ImageNet
Hayashi et al. (2022) [54]	USA	Image	Hand, Elbow, Shoulder, Foot, Leg	Detectron2
Ho-Le et al. (2017) [79]	Australia	Tabular	Hip	ANN, KNN, SVM
Inoue et al. (2022) [9]	Japan	Image	Pelvic, Spine, Rib	CNN
Kim et al. (2018) [69]	England	Image	Wrist	Inception v3 CNN model (transfer learning, trained in non-fracture images)
Kitamura et al. (2020) [55]	USA	Image	Hip	CNN
Korfiatis et al. (2018) [81]	NA	Image	Trabecular bone	Multilayer Perceptron SVM
Kruse et al. (2017) [13]	Denmark	Tabular	Hip	Twenty-four statistical models were built
Del Lama et al. (2022) [80]	Brazil	Tabular+Image	Vertebral	CNN, Multilayer Perceptron
Lemineur et al. (2007) [70]	France	Tabular	Osteoporotic Fracture	ANN
Lindsey et al. (2018) [56]	USA	Image	Wrist	CNN
Liu et al. (2015) [32]	Taiwan	Tabular	Hip	ANN
Liu et al. (2022) [48]	China	Image	Hip	CNN
Mawatari et al. (2020) [37]	Japan	Image	Hip	CNN
Mehta et al. (2020) [57]	USA	Tabular+Image	Lumbar Spine	SVM with a different kernel
Minonzio et al. (2020) [71]	France	Image	Hip	SVM, LR
Monchka et al. (2021) [58]	Canada	Image	Vertebral	CNN
Monchka et al. (2022) [59]	Switzerland, Canada	Image	Vertebral	CNN, Active Learning
Mu et al. (2021) [49]	China	Image	Femoral Neck	CNN
Murata et al. (2020) [38]	Japan	Image	Vertebral	CNN
Mutasa et al. (2020) [60]	USA	Image	Femoral Neck	CNN
Nguyen et al. (2022) [61]	USA	Image	Foot, Ankle, Knee, Leg, Hand, Wrist, Elbow, Arm, Shoulder, Clavicle	CNN
Nishiyama et al. (2014) [39]	Japan	Image	Hip	SVM
Nissinen et al. (2021) [72]	Finland	Image	Vertebral	CNN
Oakden-Rayner et al. (2022) [62]	USA, Australia	Image	Hip	CNN
Ozkaya et al. (2022) [73]	Turkey	Image	Scaphoid	CNN
Raghavendra et al. (2018) [82]	NA	Image	Thoracolumbar	CNN
Raisuddin et al. (2021) [74]	Finland	Image	Wrist	CNN
Ramos et al. (2022) [10]	Brazil	Image	Vertebral	CNN, SVM, KNN, ExtraTrees, QDA
Regnard et al. (2022) [75]	France	Image	Pelvic, Limbs	CNN
Rosenberg et al. (2022) [76]	Italy	Image	Thoracolumbar	CNN
Salehinejad et al. (2021) [83]	NA	Image	Vertebral	CNN with ResNet-50+BLSTM layer
Sato et al. (2021) [40]	Japan	Image	Hip	CNN with EfficientNet-B4 model (a pre-trained ImageNet model)
Small et al. (2021) [63]	USA	Image	Cervical Spine	CNN
Su et al. (2019) [64]	USA	Tabular	Hip	Classification and regression tree
Tomita et al. (2018) [65]	USA	Image	Vertebral	CNN
Tseng et al. (2013) [33]	Taiwan	Tabular	Hip	LR, Ensemble ANN
Ulivier et al. (2021) [77]	Italy	Tabular	Vertebral	ANN
Urakawa et al. (2019) [41]	Japan	Image	Hip	Transfer learning of CNN (VGG_16 network)
Ureten et al. (2022) [78]	Turkey	Image	Hand	Transfer Learning
Wang et al. (2022) [84]	NA	Image	Vertebral	CNN
Wu et al. (2020) [14]	USA	Tabular	Major Osteoporotic Fractures	LR, GB, RF, ANN
Yabu et al. (2021) [11]	Japan	Image	Vertebral	CNN
Yamada et al. (2020) [42]	Japan	Image	Hip	CNN
Yamamoto et al. (2020) [43]	Japan	Image	Hip	CNN
Yeh et al. (2022) [34]	Taiwan, USA	Image	Vertebral	Transfer Learning
Yi-Chu Li et al. (2021) [35]	Taiwan	Image	Vertebral	Transfer learning, Ensemble model
Yoda et al. (2022) [44]	Japan	Image	Vertebral	CNN, Transfer Learning
Yoon et al. (2021) [36]	Taiwan, USA	Image	Scaphoid	CNN
Yu et al. (2020) [66]	USA	Image	Hip	Transfer Learning
Yuan Li et al. (2021) [50]	China	Image	Vertebral	CNN (ResNet50)

CNN, Convolution Neural Network; SVM, Support Vector Machine; LR, Logistic Regression; RF, Random Forest; ANN, Artificial Neural Network; MLP, Multi Layers Perceptron; KNN, K-Nearest Neighbors; GB, Gradient Boosting; NLP, Natural Language Processing; QDA, Quadratic Discriminant Analysis

Fracture identification was performed using imaging-related data in 54 studies, tabular data in nine studies, and imaging and tabular data in three. Of the 57 studies using imaging-related and combined data, 33 analyzed radiograph images [6,7,28–31,35–38,40–42,45,47–49,52–57,59,61,62,66–68,72–74,78], 12 analyzed computed tomography (CT) images [8,9,39,43,50,63,65,69,75,81–83], and the remaining studies analyzed other imaging modalities (**S1 Table, and S2 Table**). The most common fracture outcome was vertebral fracture (20 studies) [8,10,11,28,31,34,35,38,44,46,50,51,58,59,65,72,77,80,83,84], followed by hip [6,13,29,32,33,37,39–43,48,53,62,64,66,68,79], and other fracture types (**Table 1**).

### AI algorithms summary

Among the 54 studies that utilized imaging-related data, convolutional neural networks (CNN), a deep learning approach, emerged as the predominant choice, followed by instances where transfer learning was adopted. In some cases, the limited availability of labeled image data prompted the utilization of transfer learning [53,69], and certain studies incorporated pre-trained CNNs with non-fracture-related radiological images [6,28,85]. The prevailing preference was for fully connected artificial neural networks within the subset of nine studies involving tabular data. Logistic regression and ensemble learning models were commonly employed, including Random Forest, Gradient Boosting, and XGBoost. Among the three studies that harnessed both image and tabular data, a notable trend was the adoption of the support vector machine with various kernel models [57,68].

### Handling imbalanced data and data augmentation

Imbalanced fracture outcomes were reported in 48 studies (**S3 Table**). Only 12 studies addressed the handling of imbalance outcomes during model development, using Synthetic Minority Over-sampling Technique (SMOTE) [86] or undersampling [35]. Data augmentation was frequently utilized in image studies, including horizontal and vertical rotation [45,50,58,67,69,72], adding Gaussian noise [67], random rescaling and flipping [30,53], mirroring, and lighting and contrast adjustments [56].

### Hyperparameter optimization

Thirty-six studies reported the detailed process for optimizing hyperparameters in the final selected models (**S3 Table**). Beyaz et al. utilized genetic algorithms to identify the optimal hyperparameters for their CNN architecture [67]. Liu et al. explored the impact of varying the number of hidden neurons in the output layer [32]. Nissinen et al. [72] employed two approaches for hyperparameter searches: random search [87] and hyperband [88].

### Data split and validation in an external data set

Fifty-one studies reported the split sample for model development (training) and validation (testing) (**S3 Table**). No universal rule of data separation was found. A different set of split samples was utilized, e.g., 80% training and 20% testing [10,28,47,57,71], 90% training and 10% testing [32,33,56,81], and 80% training, 10% validation, and 10% testing [40,41,65,69]. Twenty studies reported the cross-validation with 20-folds [66], 10-folds [8,14,33,34,39,45,50,53,57,64,72,76,80,81], 5-folds [13,28,32,38,44,46,48,67,74,78,79], and 7-folds [83]. Thirteen studies performed an out-of-sample external validation [6,7,29–31,35,47,49,56,59,62,72,74]. Choi et al. [47] performed external tests using two types of distinct datasets: temporal data, which was obtained at a different period from the model development, and other geographically separated data, which was collected from a different center. Li et al. [35] utilized a dataset from another medical center that used a different plain radiographic technique.

### Meta-analysis

**Fig 2:**
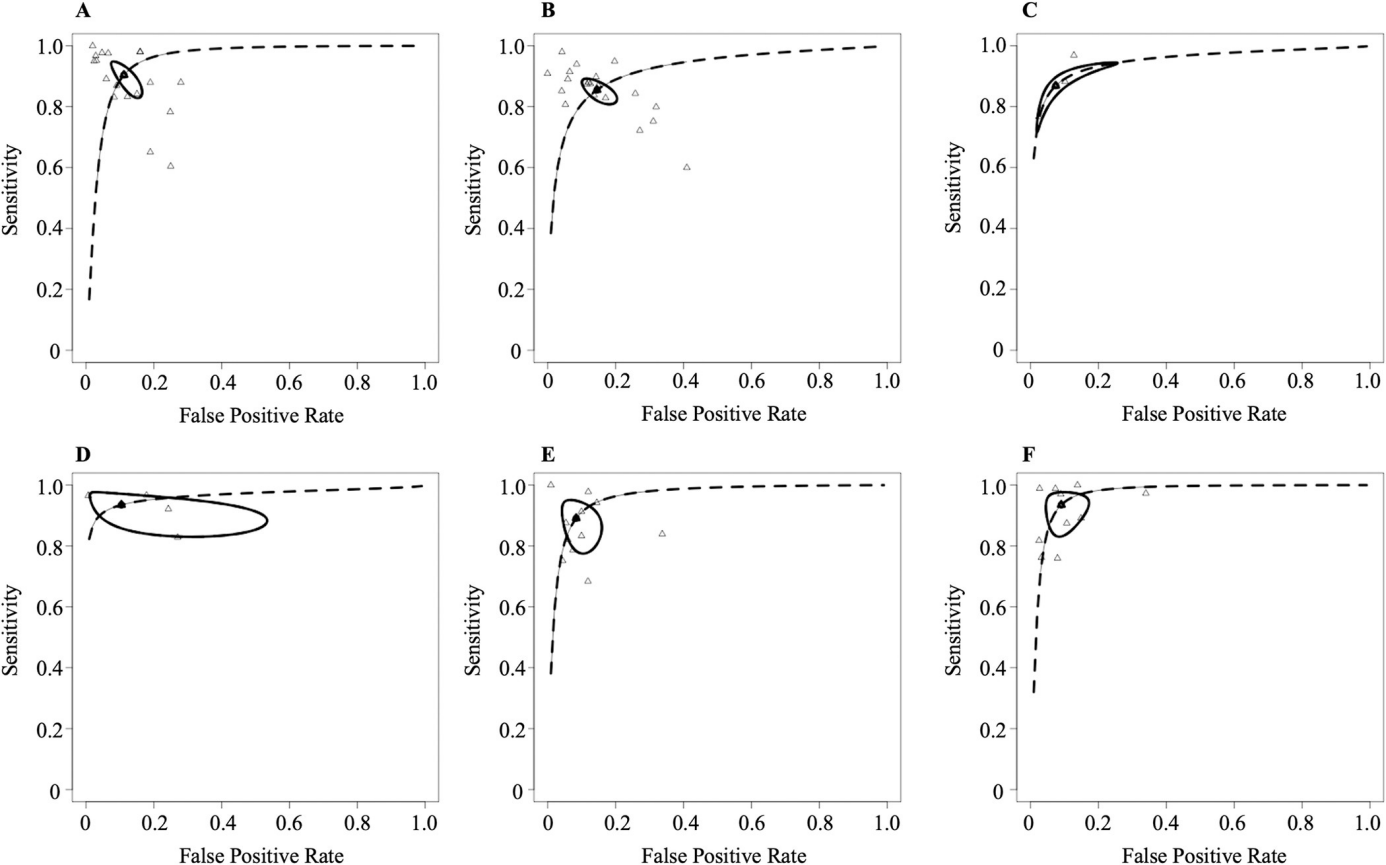
The hierarchical summary receiver operating characteristic curve for different fracture types in the meta-analysis. **A**: Hip (18 studies), **B**: Vertebral (20 studies), **C**: Wrist (3 studies), **D**: Femoral Neck (4 studies), **E**: Multiple (11 studies), and **F**: Others (10 studies).

We extracted 66 contingency tables for each selected study (**S4 Table**). The overall pooled sensitivity and specificity, calculated using logit transformation, were 91% (95% CI: 88, 93) and 90% (95% CI: 88, 92), respectively (**Table 2**). The pooled sensitivities for hip and vertebral fractures were found to be 92% (95% CI: 87–96) and 86% (95% CI: 82–89), respectively, while the pooled specificities for these fractures were 90% (95% CI: 85–93) and 86% (95% CI: 81–90), respectively (**Table 2**). The unified hierarchical summary receiver operating characteristic curve for different fracture types is shown in **Fig 2**. The area under the curve (AUC) was highest for femoral neck fractures at 0.98, followed by other fractures (0.97), multiple fractures (0.93), hip fractures (0.91), wrist (0.86), and vertebral (0.84).

**Table 2:** Pooled Sensitivities, Specificities, and Diagnostic Odds Ratio for 60 studies in different fractures outcome. Studies with only one selected fracture outcome (cervical spine, hand, lumber spine, proximal humerus, supracondylar, and trabecular bone) were omitted.

Outcome	Sensitivity (%)^1)^	Specificity (%)^1)^	Sensitivity (%)^2)^	Specificity (%)^2)^	Diagnostic Odds Ratio	No. of Studies included
Overall	0.91 (0.88, 0.93)	0.90 (0.88, 0.92)	0.89 (0.87, 0.91)	0.88 (0.86, 0.91)	81.14 (53.69, 122.63)	66
Vertebral	0.86 (0.82, 0.89)	0.86 (0.81, 0.90)	0.86 (0.82, 0.89)	0.86 (0.81, 0.90)	38.26 (21.36, 68.51)	20
Hip	0.92 (0.87, 0.96)	0.90 (0.85, 0.93)	0.90 (0.85, 0.95)	0.89 (0.85, 0.93)	99.50 (39.37, 251.48)	18
Multiple*	0.90 (0.81, 0.96)	0.92 (0.87, 0.95)	0.88 (0.81, 0.94)	0.91 (0.85, 0.95)	88.71 (33.54, 234.64)	11
Femoral Neck	0.94 (0.87, 0.97)	0.90 (0.64, 0.98)	0.93 (0.86, 0.98)	0.85 (0.68, 0.97)	125.82 (10.96, 1444.74)	4
Wrist	0.90 (0.76, 0.96)	0.93 (0.85, 0.97)	0.89 (0.75, 0.97)	0.93 (0.85, 0.98)	105.68 (56.44, 197.89)	3
Scaphoid	0.92 (0.68, 0.98)	0.81 (0.54, 0.94)	0.89 (0.61, 1.00)	0.80 (0.49, 0.98)	65.27 (44.16, 96.46)	2
Thoracolumbar	0.97 (0.84, 0.99)	0.92 (0.90, 0.95)	0.95 (0.80, 1.00)	0.92 (0.90, 0.95)	278.30 (15.99, 4843.58)	2

Data in parentheses are 95% confidence intervals.

^1)^: the logit transformation was used to calculate the pooled sensitivity and specificity.

^2)^: the arcsine transformation was used to calculate the pooled sensitivity and specificity.

* Multiple fractures outcome studies include hip and pelvic (2), hip and spine (1), major osteoporotic fractures (1), multiple (3), osteoporotic fractures (2), pelvic and limbs (1), pelvic, spine, and rib (1).

### Sensitivity analysis

Arcsine transformation yielded similar results with the pooled sensitivity at 89% (95% CI: 87, 91) and specificity at 88% (95% CI: 86, 91). Among data types, studies using only image data exhibited superior diagnostic performance with sensitivity and specificity at 91% (95% CI: 88, 93) and 89% (95% CI: 78, 91) using the arcsine transformation (**Table 3**). Studies employing radiographs displayed the highest sensitivity (92% [95% CI: 89, 95]) and specificity (90% [95% CI: 87, 93]) using the arcsine transformation (**Table 4**).

**Table 3:** Pooled Sensitivities, Specificities, and Diagnostic Odds Ratio for 66 studies in different data type used.

Data Type	Sensitivity (%)^1)^	Specificity (%)^1)^	Sensitivity (%)^2)^	Specificity (%)^2)^	Diagnostic Odds Ratio	No. of Studies included
Tabular	0.81 (0.77, 0.85)	0.83 (0.76, 0.88)	0.81 (0.76, 0.85)	0.82 (0.76, 0.87)	20.06 (12.14, 33.16)	9
Image	0.92 (0.90, 0.94)	0.91 (0.88, 0.93)	0.91 (0.88, 0.93)	0.89 (0.87, 0.91)	104.20 (65.12, 166.72)	54
Tabular + Image	0.84 (0.76, 0.89)	0.95 (0.88, 0.98)	0.84 (0.77, 0.90)	0.96 (0.89, 1.00)	73.15 (27.23, 196.52)	3

Data in parentheses are 95% confidence intervals.

^1)^: the logit transformation was used to calculate the pooled sensitivity and specificity.

^2)^: the arcsine transformation was used to calculate the pooled sensitivity and specificity.

**Table 4:** Pooled sensitivities, specifications, and diagnostic odds ratios for 54 studies (including three from the tabular and image data used) in different image modalities. Studies with only one selected image modality (Radiograph + CT + MRI, Radiograph + MRI, UGWSI) were omitted.

Image Modality	Sensitivity (%)^1)^	Specificity (%)^1)^	Sensitivity (%)^2)^	Specificity (%)^2)^	Diagnostic Odds Ratio	No. of Studies included
CT	0.89 (0.80, 0.94)	0.90 (0.85, 0.93)	0.86 (0.79, 0.92)	0.89 (0.84, 0.93)	67.16 (28.34, 159.18)	12
MRI	0.91 (0.83, 0.95)	0.89 (0.84, 0.93)	0.91 (0.84, 0.96)	0.91 (0.84, 0.95)	89.46 (26.41, 302.99)	5
Radiograph	0.94 (0.90, 0.96)	0.92 (0.89, 0.94)	0.92 (0.89, 0.95)	0.90 (0.87, 0.93)	150.92 (76.75, 296.78)	33
Radiograph + CT	0.93 (0.79, 0.98)	0.84 (0.81, 0.87)	0.92 (0.75, 1.00)	0.84 (0.80, 0.88)	66.11 (16.48, 265.26)	2
VFAI	0.87 (0.86, 0.89)	0.88 (0.87, 0.89)	0.87 (0.86, 0.89)	0.88 (0.87, 0.89)	50.64 (42.14, 60.86)	2

Data in parentheses are 95% confidence intervals.

^1)^: the logit transformation was used to calculate the pooled sensitivity and specificity.

^2)^: the arcsine transformation was used to calculate the pooled sensitivity and specificity.

UGWSI: Ultrasonic Guided Wave Spectrum Image, VFAI: Vertebral Fracture Assessment Image

### Subgroup analysis

**Fig 3:**
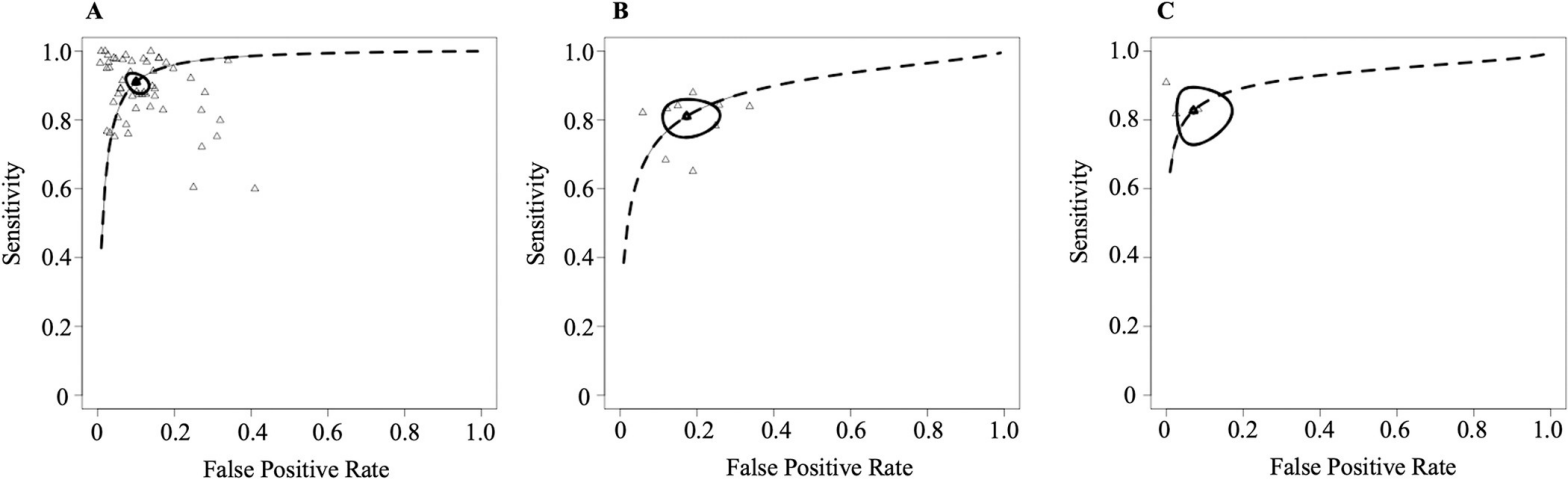
Unified hierarchical summary receiver operating characteristic curve for different data types in the meta-analysis. **A**: image (54 studies), **B**: tabular (9 studies), and **C**: image and tabular (3 studies).

Among data types, studies using only image data exhibited superior diagnostic performance with sensitivity and specificity at 92% (95% CI: 90, 94) and 91% (95% CI: 88, 93), respectively, when using logit transformation (**Table 3**). Studies employing radiographs displayed the highest sensitivity (94% [95% CI: 90, 96]) and specificity (92% [95% CI: 89, 94]) using logit transformation (**Table 4**). The AUC for radiograph studies (0.94) was higher than studies using radiograph and CT together (0.89) or MRI alone (0.88). The diagnostic odds ratio (DOR) was highest for hip fractures at 99.50 (95% CI: 39.37, 251.48) compared to vertebral fractures (38.26 [95% CI: 21.36, 68.51]) (**Table 2**). The AUC for image data studies (0.96) was higher than that for those using tabular and images together (0.83) or tabular data alone (0.81) (**Fig 3**).

### Publication bias

The assessment of publication bias encompassed each fracture outcome and the utilization of distinct data types (**S5 and S6 Tables, S1–S3 Figs**). The Contour-Enhanced Funnel Plot illustrated the study distribution, and its enhanced contour facilitated the identification of potential bias (**S1—S3 Figs)**. Notably, asymmetrical distribution was evident in the context of hip and vertebral fracture outcomes, and the studies used image data only (**S1 Fig and S3 Fig**). This asymmetry implies the presence of possible publication bias, particularly pronounced in studies with smaller sample sizes. However, the trim-and-fill method corrected this asymmetry, rendering the distribution symmetrical (**S2 Fig and S3 Fig**). After using the trim-and-fill method to adjust for publication bias, the diagnostic odds ratio (DOR) has revealed that the effect size remains statistically significant (**S5 and S6 Tables**).

### Risk of bias and applicability

**Fig 4:**
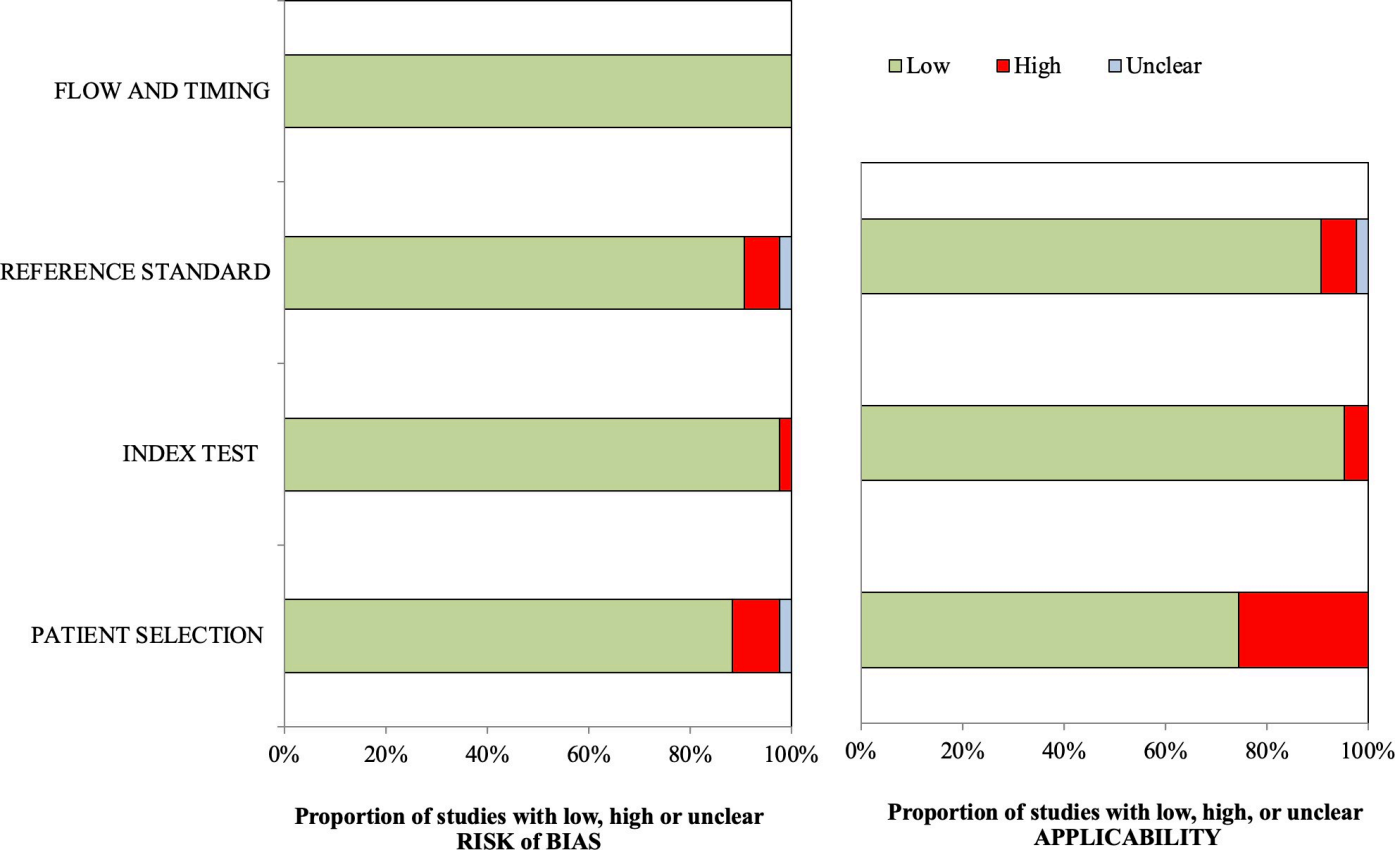
Summary of the Quality Assessment of Diagnostic Accuracy Studies for the risk of bias and applicability in the included 66 studies. The risk of bias was measured in four domains: patient selection, index test, reference standard, and flow and timing. The risk of applicability was evaluated with three domains: patient selection, index test, and reference.

The assessment of bias and applicability for 66 studies revealed moderate to low concerns (**Table 5** and **Fig 4**). Patient selection and reference standards were the primary concerns for bias and applicability. Many studies lacked the reporting of sample characteristics such as gender and age, limiting generalizability. Some studies did not report patient selection or reference standard computation methods [62,75,78]. Threshold adjustments in some studies might have led to overfitting, reducing the generalizability of the models [72]. Most studies exhibited applicability concerns and needed to be more easily generalizable to other populations. For example, one study [66] focused on patients visiting the emergency department for acute proximal femoral fracture, limiting generalizability to the general population. Another study included patients with existing vertebral fractures, reducing generalizability to the general population. Data preprocessing often involves the removal of occult fractures, with some studies excluding radiographic occult fractures requiring additional modalities for confirmation [53]. Other studies excluded images with uncertain, traumatic, or pathological fractures or those with insufficient quality or resolution [58]. A few studies did not provide specific locations for fracture types or specify which ones were included [12,70].

**Table 5:** The result of methodological quality for 66 included studies in the assessment of the risk of bias and applicability.

First author (Year published)	RISK OF BIAS				APPLICABILITY CONCERNS		
	PATIENT SELECTION	INDEX TEST	REFERENCE STANDARD	FLOW AND TIMING	PATIENT SELECTION	INDEX TEST	REFERENCE STANDARD
Almog et al. (2020) [12]	+	+	+	+	+	+	+
Bae et al. (2021) [7]	+	+	+	+	+	+	+
Beyaz et al. (2020) [67]	+	+	+	+	+	+	+
Burns et al. (2017) [8]	−	+	+	+	−	−	+
Chen et al. (2021) [28]	+	+	+	+	+	+	+
Chen et al. (2022) [46]	+	+	+	+	+	+	+
Cheng et al. (2019) [6]	+	+	+	+	+	+	+
Cheng et al. (2020) [29]	+	+	+	+	+	+	+
Cheng et al. (2021) [30]	+	+	+	+	+	+	+
Choi et al. (2020) [47]	+	+	+	+	−	+	+
Chou et al. (2022) [31]	+	+	+	+	+	+	+
Chung et al. (2018) [45]	+	+	−	+	−	+	−
Derkatch et al. (2019) [51]	+	+	+	+	+	+	+
Galassi et al. (2020) [68]	+	+	+	+	+	+	+
Guermazi et al. (2022) [52]	+	+	+	+	+	+	+
Gupta et al. (2020) [53]	+	+	+	+	+	+	+
Hayashi et al. (2022) [54]	+	+	+	+	−	+	+
Ho-Le et al. (2017) [79]	−	+	+	+	+	+	+
Inoue et al. (2022) [9]	+	+	+	+	−	+	+
Kim et al. (2018) [69]	+	+	+	+	+	+	+
Kitamura et al. (2020) [55]	+	+	+	+	+	+	+
Korfiatis et al. (2018) [81]	+	+	+	+	−	+	+
Kruse et al. (2017) [13]	+	+	+	+	+	+	+
Del Lama et al. (2022) [80]	+	+	+	+	+	+	+
Lemineur et al. (2007) [70]	+	+	−	+	−	+	−
Lindsey et al. (2018) [56]	+	+	+	+	+	+	+
Liu et al. (2015) [32]	+	+	+	+	+	+	+
Liu et al. (2022) [48]	+	+	+	+	+	+	+
Mawatari et al. (2020) [37]	+	+	+	+	+	+	+
Mehta et al. (2020) [57]	+	+	+	+	−	+	+
Minonzio et al. (2020) [71]	+	−	+	+	+	−	+
Monchka et al. (2021) [58]	O	+	+	+	−	+	+
Monchka et al. (2022) [59]	+	+	+	+	+	+	+
Mu et al. (2021) [49]	+	+	+	+	+	+	+
Murata et al. (2020) [38]	+	+	+	+	+	+	+
Mutasa et al. (2020) [60]	+	+	+	O	+	+	O
Nguyen et al. (2022) [61]	+	+	+	+	+	+	+
Nishiyama et al. (2014) [39]	O	+	+	+	+	+	+
Nissinen et al. (2021) [72]	+	+	O	+	O	+	O
Oakden-Rayner et al. (2022) [62]	+	+	−	+	O	+	−
Ozkaya et al. (2022) [73]	+	+	+	+	+	+	+
Raghavendra et al. (2018) [82]	O	+	+	+	O	+	+
Raisuddin et al. (2021) [74]	+	+	+	+	+	+	+
Ramos et al. (2022) [10]	+	+	+	+	+	+	+
Regnard et al. (2022) [75]	−	+	+	+	−	+	+
Rosenberg et al. (2022) [76]	+	+	+	+	+	+	+
Salehinejad et al. (2021) [83]	+	+	+	+	+	+	+
Sato et al. (2021) [40]	+	+	+	+	+	+	+
Small et al. (2021) [63]	+	+	+	+	+	+	+
Su et al. (2019) [64]	+	+	+	+	+	+	+
Tomita et al. (2018) [65]	+	+	+	+	+	+	+
Tseng et al. (2013) [33]	+	+	+	+	+	+	+
Ulivier et al. (2021) [77]	+	+	+	+	+	+	+
Urakawa et al. (2019) [41]	+	+	+	+	+	+	+
Ureten et al. (2022) [78]	−	+	+	+	−	+	+
Wang et al. (2022) [84]	+	+	+	+	+	+	+
Wu et al. (2020) [14]	+	+	+	+	+	+	+
Yabu et al. (2021) [11]	+	+	+	+	+	+	+
Yamada et al. (2020) [42]	+	+	+	+	+	+	+
Yamamoto et al. (2020) [43]	+	+	+	+	+	+	+
Yeh et al. (2022) [34]	+	−	+	+	+	+	+
Yi-Chu Li et al. (2021) [35]	+	+	+	+	+	+	+
Yoda et al. (2022) [44]	+	+	+	+	+	+	+
Yoon et al. (2021) [36]	+	+	+	+	+	+	+
Yu et al. (2020) [66]	+	+	+	+	+	+	+
Yuan Li et al. (2021) [50]	+	+	+	+	+	+	+

+: Low risk of bias/no concerns regarding applicability, −: High risk of bias/concerns regarding applicability, O: Unclear risk of bias/unclear whether there are concerns regarding applicability.

## Discussion

Our systematic review and meta-analysis offer the most current and comprehensive evaluation of the diagnostic accuracy of Artificial Intelligence (AI) for predicting various osteoporotic fracture outcomes using various imaging modalities and data types. This study represents the first systematic review and quantitative meta-analysis of AI’s diagnostic accuracy and comparison using different data types across multiple fracture outcomes. Our analysis reveals four major findings. First, AI provides high classification accuracy for fracture detection when utilizing imaging data, with a pooled sensitivity of 92% (95% CI: 90, 94). Convolutional neural networks with transfer learning exhibit significantly high accuracy when using image data in classifying fractures. Second, our study comprehensively reviews diagnostic accuracy among different image modalities with AI. While all image modalities provide comparable results, AI with radiograph images yields the highest results with a pooled sensitivity of 94% (95% CI: 90, 96). Third, our sensitivity analysis, employing the arcsine transformation, which was complemented by the primary analysis utilizing the logit transformation, provides the robustness of our findings. Both methodologies yielded similar results regarding pooled sensitivity and specificity, which underscores the reliability and consistency of our findings. Fourth, significant flaws were observed in the study design and reporting of AI for real-world applicability. For example, only a few studies described the patient characteristics of data, and only half (n = 33) reported the hyperparameter selection process.

Our findings align with other systematic reviews and meta-analyses [15,16], showing that AI demonstrates considerably higher pooled sensitivity and specificity. However, inconsistent results have been observed when comparing different image modalities in fracture detection. External validation enables a more robust demonstration of clinical utility versus simple internal train/test cross-validation. Our study shows that only thirteen studies (20%) out of sixty-six performed external validation. The limitation of validating in an external dataset is the lack of availability of large, labeled datasets due to resistance to sharing data across institutions because of patient privacy issues and the necessity of experts for labeling the datasets. Although external validation enhances the robustness of AI systems, it could potentially attenuate their impact on the system. Consequently, it’s crucial to acknowledge that external validation might not always be advisable due to the potential impact of factors like sample size and the diversity of the training set. Two systematic reviews [89,90] provide valuable insights into the current limitations of AI studies. A broad discussion of possible solutions is necessary because methodological challenges, risk of bias, and applicability concerns can arise in AI during all stages of development, including data curation, model selection, implementation, and validation. Both reviews recommend that researchers follow standardized reporting guidelines to determine the risk of bias and improve methodological quality assessment.

Our study has limitations; the major one is that only a few studies that employed tabular data or combined tabular and image data are eligible. Second, we excluded non-English-language articles, which may have overlooked some studies published in a different language. Third, many of these included studies had study design flaws. They were classified as having great concern for bias and applicability, limiting the conclusions that could be drawn from the meta-analysis because studies with a high risk of bias and applicability overestimated algorithm performance.

This systematic review and meta-analysis have important implications for clinical practice. Given the high diagnostic performance of AI, these techniques could be integrated into existing fracture risk assessment tools to enhance the identification of patients at risk and facilitate early intervention. Healthcare professionals should be trained in interpreting and applying these methods in clinical practice.

This study observed superior prediction performance with single radiograph input data over multimodal imaging, which can be attributed to the radiographs’ consistent and standardized anatomical view, reducing noise and variability inherent in multimodal inputs [91]. Radiographs precisely capture fracture-relevant features, while added modalities like CT and MRI can diversify and possibly weaken these key features [92]. Multimodal inputs can also elevate overfitting risks, particularly with limited datasets [93]. Radiographs, being more accessible and cost-effective than CT or MRI, allow for larger, representative datasets enhancing model performance. The decision between single radiographs and multimodal inputs should be rooted in the research context, data availability, and prediction objectives. Despite the evident advantages of radiographs, specific scenarios may warrant multimodal integration for improved predictions. We also observed that solely relying on image data produced better AUC values than combining it with tabular data. Image data’s richness and direct relevance to fracture detection offer clear diagnostic advantages [94]. Convolutional neural networks (CNNs), identified in our study, are adept at processing this data, emphasizing subtle fracture-related visual nuances [95]. In contrast, tabular data could infuse noise and inconsistencies. Sole image data ensures focus on vital visual features and offers a more standardized data format than diverse tabular inputs.

Further research is needed to address the limitations identified in the included studies and to explore the performance of specific ML and DL algorithms. Researchers should provide more detailed information about their study populations and methods, including patient selection, fracture type location, and the reference standard used. Future studies should also investigate the impact of factors such as training dataset size, model architecture, and the inclusion of clinical and demographic variables on the diagnostic performance of AI. Future research will help develop more accurate and generalizable models for predicting osteoporotic fractures and inform evidence-based clinical practice. Several novel diagnostic meta-analysis methodologies have recently been introduced [96–98]. Nevertheless, due to the limited sample sizes within selected studies focusing on fractures beyond vertebral and hip injuries and studies involving tabular and tabular and image data types, incorporating these methodologies into our present study was unfeasible. While we acknowledge their potential applicability, the current study’s unique characteristics led us to refrain from their implementation. We will implement these methodologies in our forthcoming investigations, particularly as more comprehensive studies become available. In aid of future researchers, we provide an array of crucial challenges and their potential resolutions pertinent to applying machine learning or deep learning for fracture diagnosis (**S7 Table**).

In conclusion, our meta-analysis highlights the high diagnostic accuracy of AI in various fracture outcomes. As AI demonstrates reliable results in fracture detection, it holds the potential to streamline fracture diagnosis in healthcare systems. However, transparent reporting of study methods and designs for AI development and validation is essential to ensure their real-world applicability. By addressing the current research landscape’s limitations and promoting standardized guidelines, we can facilitate the integration of AI technologies into clinical practice and enhance the prediction of osteoporotic fractures, ultimately leading to improved patient care.

## Supporting information

S1 PRISMA Checklist PRISMA DTA Checklist. (DOCX)

S1 Text The search term used for each engine: 1) PubMed, 2) Web of Science, and 3) IEEE. (DOCX)

S1 Table A characteristic of 57 selected studies for Image modality, Image Data Type, and Data Source. (DOCX)

S2 Table The data source of 9 selected studies used tabular data, and 3 studies (in bold) used both tabular and image data. (DOCX)

S3 Table A characteristic of 66 selected studies for the unbalanced outcome, a technique used for an unbalanced outcome, data preprocessing, hyperparameters optimization, and performance measurement used. (DOCX)

S4 Table A summary of the contingency table for 66 selected studies. (DOCX)

S5 Table Summary of Publication Bias Assessment across different fracture outcomes. TF: Trim and Fill method, DOR: Diagnostic Odds Ratio, CI: Confidence Interval.
(DOCX)

S6 Table Summary of Publication Bias Assessment across different data types. TF: Trim and Fill method, DOR: Diagnostic Odds Ratio, CI: Confidence Interval.
(DOCX)

S7 Table Overview of Key Challenges and Potential Resolutions in the Utilization of Machine Learning or Deep Learning for Fracture Diagnosis. (DOCX)

S1 Fig Contour-Enhanced Funnel Plot for Publication Bias Assessment across Different Fracture Outcomes. (DOCX)

S2 Fig Contour-Enhanced Funnel Plot for Publication Bias Assessment across Different Fracture Outcomes after Employing the Trim & Fill Method. The open circle represents the “filled” studies from the Trim & Fill Method in each fracture outcome plot.
(DOCX)

S3 Fig Contour-Enhanced Funnel Plot: Evaluating Publication Bias Across Various Data Types. The top row illustrates the funnel plot encompassing all studies. The second row shows the Contour-Enhanced Funnel Plot for Publication Bias Assessment after employing the Trim & Fill Method. The open circle designates the studies “filled” through the Trim & Fill Method within each contour-enhanced funnel plot in the second row.
(DOCX)
