# Supplementary material for: Artificial intelligence in fracture detection with different image modalities and data types: A systematic review and meta-analysis
Source: PLOS Digit Health. 2024 Jan 30;3(1):e0000438. doi: 10.1371/journal.pdig.0000438 (PMC10826962; doi:10.1371/journal.pdig.0000438)
Supplement: S1 Text — (DOCX) [file pdig.0000438.s002.docx]

We show the search term used for each engine: 1) PubMed, 2) Web of Science, and 3) IEEE, and the last of these searches were performed on December 15, 2022.

1. PubMed: resulted in 317 studies

("Osteoporosis"[Mesh] OR "Fractures, Bone"[Mesh:noexp] OR "Osteoporotic Fractures"[Mesh] OR "Hip Fractures"[Mesh] OR "Humeral Fractures"[Mesh] OR "Spinal Fractures"[Mesh] OR "Bone Density"[Mesh] OR Osteoporo*[tiab] OR "fragility fracture*"[tiab] OR (Fracture*[tiab] AND (humer*[tiab] OR spin*[tiab] OR vertebra*[tiab] OR hip[tiab] OR forearm[tiab])) OR "bone densit*"[tiab] OR "bone mineral densit*"[tiab]) AND ("Artificial Intelligence"[Mesh:noexp] OR "machine learning"[Mesh] OR "Neural Networks, Computer"[Mesh] OR "artificial Intelligence"[tiab] OR "machine learning"[tiab] OR "deep learning"[tiab] OR "neural network*"[tiab]) AND English[la]

1. Web of Science: resulted in 385 studies

TS=((Osteoporo* OR (Fracture* NEAR/3 (humer* OR spin* OR vertebra* OR “hip” OR “forearm” OR “fragility”) ) OR (“bone” NEAR/3 densit*) ) AND (“artificial Intelligence” OR “machine learning” OR “deep learning” OR “neural network*”) )

1. IEEE: resulted in 426 studies

(“Document Title”: “machine learning” OR “artificial intelligence” OR “neural network” OR “CNN” OR “artificial neural network” OR “multilayer perceptron” OR “ANN” OR “random forest” OR “SVM” OR “support vector machine” OR “regression” OR “logistic” OR ““lasso”) AND (“Abstract”: "roc" OR "area under the curve" OR "AUC" OR "sensitivity" OR "specificity" OR "accuracy" OR "goodness of fit" OR "performance") AND ("Document Title": “bone” OR “fracture” OR “osteoporotic” OR “bone loss” “BMD” OR “bone density”) AND (“Keywords”: “bone” OR “fracture” OR “osteoporotic” OR “bone loss” “BMD” OR “bone density”)
