# Supplementary material for: Artificial intelligence in fracture detection with different image modalities and data types: A systematic review and meta-analysis
Source: PLOS Digit Health. 2024 Jan 30;3(1):e0000438. doi: 10.1371/journal.pdig.0000438 (PMC10826962; doi:10.1371/journal.pdig.0000438)
Supplement: S1 Table — (DOCX) [file pdig.0000438.s003.docx]

**S1 Table**. A characteristic of 57 selected studies for Image modality, Image Data Type, and Data Source. NA, Not Available

| **Author** | **Image Modality** | **Image Data Type** | **Data Source** |
| --- | --- | --- | --- |
| Bae et al. (2021) | Radiograph | Plain Pelvic Radiographs | Two tertiary hospitals (Seoul and Gyeonggi-do) |
| Beyaz et al. (2020) | Radiograph | Radiographs | Başkent University Adana Turgut Noyan Training and Research Centre |
| Burns et al. (2017) | CT | CT | University of California Irvine medical center |
| Chen et al. (2021) | Radiograph | Radiographs (Plain abdominal frontal) | National Taiwan University Hospital (2015 - 2018) |
| Chen et al. (2022) | Radiograph + MRI | MRI, Digital radiography | The Second Affiliated Hospital of Chongqing Medical University |
| Cheng et al. (2019) | Radiograph | Plain frontal pelvic radiographs | Chang Gung Memorial Hospital (CGMH) |
| Cheng et al. (2020) | Radiograph | Plain pelvic radiographs | Chang Gung Memorial Hospital (CGMH) |
| Cheng et al. (2021) | Radiograph | Radiographs | Chang Gung Memorial Hospital (CGMH) |
| Choi et al. (2020) | Radiograph | Radiographs (anteroposterior and lateral elbow) | Seoul National University Hospital and Gyeongsang National University Changwon Hospital |
| Chou et al. (2022) | Radiograph | Plain Pelvic Radiographs | National Yang Ming Chiao Tung Univeristy |
| Chung et al. (2018) | Radiograph | Plain anteroposterior shoulder radiographs | Konkuk University Medical Center; Kyungpook National University Hospital; Myungji Hospital; Kangwon National University Hospital; National Police Hospital; Seoul Saint Mary’s Hospital; Wonkwang University Sanbon Hospital |
| Derkatch et al. (2019) | Vertebral fracture assessment images | Vertebral fractures assessment images | Manitoba BMD Program |
| Galassi et al. (2020) | Radiograph | Clinical, Geometric, and biomechanical variables | Hospital Mútua Terrassa; CETIR Medical Group |
| Guermazi et al. (2022) | Radiograph | Plain Pelvic Radiographs | 22 institutions in the USA |
| Gupta et al. (2020) | Radiograph | Anteroposterior radiographs | Emergency Department (ED) |
| Hayashi et al. (2022) | Radiograph | Plain Pelvic Radiographs | NA |
| Inoue et al. (2022) | CT | CT | Chiba University |
| Kim et al. (2018) | CT | CT | Royal Devon & Exeter Hospital |
| Kitamura et al. (2020) | Radiograph | Radiographs | University of Pittsburgh Medical Center |
| Korfiatis et al. (2018) | CT | CT | European project Living Human Digital Library (LHDL) and MOSAIC project |
| Lama et al. (2022) | MRI | MRI | University of Sao Paulo |
| Lindsey et al. (2018) | Radiograph | Radiographs from the posterior-anterior or lateral wrist views and other parts of the body | Hospital for Special Surgery |
| Liu et al. (2022) | Radiograph | X-ray | Five Chinese triple-A grade hospitals (Wuhan Union Hospital, Wuhan Puai Hospital, The Second Xiangya Hospital of Central South University, Xiangya Changde Hospital, and Northern Jiangsu People‚Äôs Hospital) |
| Mawatari et al. (2020) | Radiograph | Radiograph, CT, MRI | NA |
| Mehta et al. (2020) | Radiograph | Ancillary data from routine posterior-anterior | A tertiary care academic healthcare center |
| Minonzio et al. (2020) | Ultrasonic Guided Wave Spectrum Image | Ultrasonic Guided Wave Spectrum Image | Cochin Hospital |
| Monchka et al. (2021) | Vertebral fracture assessment images | Vertebral fracture assessment (Single Energy mode and Dual-energy mode) | Manitoba Bone Mineral Density Registry |
| Monchka et al. (2022) | Radiograph | Plain Pelvic Radiographs | OsteoLaus Study, Manitoba Bone Mineral Density Program, Canadian Longitudinal Study on Aging |
| Mu et al. (2021) | Radiograph | Plain Pelvic Radiographs | Three institutions’ Picture Archiving and Communication Systems were identified through the Radiology Information System |
| Murata et al. (2020) | Radiograph | Plain thoracolumbar radiography | Tokyo Medical University Hospital |
| Mutasa et al. (2020) | Radiograph + CT | Anteroposterior radiographs + CT | Columbia University Irving Medical Center |
| Nguyen et al. (2022) | Radiograph | Plain Pelvic Radiographs | Anonymized digital radiographic images from a United States-based data provider |
| Nishiyama et al. (2014) | CT | Quantitative Computed Tomography | Nagasaki University Hospital and the National Center for Geriatric and Gerontology |
| Nissinen et al. (2021) | Radiograph | Dual-Energy X-Ray Absorptiometry | Kuopio Osteoporosis Risk Factor Prevention Study, OSTPRE Fracture Prevention Study |
| Oakden-Rayner et al. (2022) | Radiograph | Plain Pelvic Radiographs | Royal Adelaide Hospital |
| Ozkaya et al. (2022) | Radiograph | Plain Pelvic Radiographs | NA |
| Raghavendra et al. (2018) | CT | CT | NA |
| Raisuddin et al. (2021) | Radiograph | Plain Pelvic Radiographs | Oulu University Hospital’s Picture Archiving and Communication System and the Radiology Information System |
| Ramos et al. (2022) | MRI | MRI | The database was acquired at the Ribeirao Preto Medical School |
| Regnard et al. (2022) | CT | CT | 14 centers in France, including 3 private hospitals, 1 public hospital, and 10 medical centers. |
| Rosenberg et al. (2022) | Radiograph + CT + MRI | Plain Pelvic Radiographs, CT, MRI | Traumatic vertebral fractures from 2010 to 2020 in a Spine Surgery Reference Center (ASST Grande Ospedale Metropolitano Niguarda, Milano, Italy) |
| Salehinejad et al. (2021) | CT | CT | NA |
| Sato et al. (2021) | Radiograph | Antero-posterior radiographs | Gamagori City Hospital, Tsushima City Hospital, Nagoya Daini Red Cross Hospital |
| Small et al. (2021) | CT | CT | NA |
| Tomita et al. (2018) | CT | CT | Dartmouth-Hitchcock Medical Center (DHMC; Lebanon, NH) |
| Urakawa et al. (2019) | Radiograph | Hip plain radiographs | Department of Orthopedic Surgery, Tsuruoka Municipal Shonai Hospital surgical registry database |
| Ureten et al. (2022) | Radiograph | Plain Pelvic Radiographs | Faculty of Medicine emergency department at Kirikkale University |
| Wang et al. (2022) | Radiograph + CT | Plain Pelvic Radiographs, CT | Two open sources of data sets |
| Yabu et al. (2021) | MRI | MRI | NA |
| Yamada et al. (2020) | Radiograph | Antero-posterior and lateral hip radiographs | Seirei Sakura Citizen Hospital; the Oyumino Central Hospital |
| Yamamoto et al. (2020) | CT | CT | Steel Memorial Hirohata Hospital |
| Yeh et al. (2022) | MRI | MRI | The radiological reporting system in a period of 4 years |
| Yi-Chu Li et al. (2021) | Radiograph | Plain lateral spine radiographs | National Chiao Tung University |
| Yoda et al. (2022) | MRI | MRI | Chiba University Hospital |
| Yoon et al. (2021) | Radiograph | Plain Pelvic Radiographs | Chang Gung Memorial Hospital (Taipei, Taiwan) and Michigan Medicine (Ann Arbor) |
| Yu et al. (2020) | Radiograph | Radiograph | NA |
| Yuan Li et al. (2021) | CT | CT | Peking University Third Hospital |
