# Supplementary material for: Artificial intelligence in fracture detection with different image modalities and data types: A systematic review and meta-analysis
Source: PLOS Digit Health. 2024 Jan 30;3(1):e0000438. doi: 10.1371/journal.pdig.0000438 (PMC10826962; doi:10.1371/journal.pdig.0000438)
Supplement: S2 Table — (DOCX) [file pdig.0000438.s004.docx]

**S2 Table**. The data source of 9 selected studies used tabular data, and 3 studies (in bold) used both tabular and image data.

| **Author** | **Data Source** |
| --- | --- |
| Almog et al. (2020) | Optum (The bone health subset, The pan-therapeutic data) |
| Ho-Le et al. (2017) | Dubbo Osteoporosis Epidemiology Study |
| **Galassi et al. (2020)** | Hospital Mútua Terrassa; CETIR Medical Group |
| Kruse et al. (2017) | National Board of Health in Denmark |
| **Lama et al. (2022)** | University of Sao Paulo |
| Lemineur et al. (2007) | Orleans Hospital |
| Liu et al. (2015) | National Taiwan University Hospital |
| **Mehta et al. (2020)** | A tertiary care academic healthcare center |
| Su et al. (2019) | Osteoporotic Fractures in Men |
| Tseng et al. (2013) | National Taiwan University Hospital |
| Ulivier et al. (2021) | A longitudinal multicentric study conducted at Fondazione IRCCS Ca’ Granda Ospedale Maggiore Policlinico of Milan, IRCCS Istituto Ortopedico Galeazzi in Milan, and IRCCS Policlinico San Donato, San Donato Milanese, Italy |
| Wu et al. (2020) | Osteoporotic Fractures in Men |
