# Supplementary material for: Artificial intelligence in fracture detection with different image modalities and data types: A systematic review and meta-analysis
Source: PLOS Digit Health. 2024 Jan 30;3(1):e0000438. doi: 10.1371/journal.pdig.0000438 (PMC10826962; doi:10.1371/journal.pdig.0000438)
Supplement: S3 Table — (DOCX) [file pdig.0000438.s005.docx]

**S3 Table**. A characteristic of 66 selected studies for the unbalanced outcome, a technique used for an unbalanced outcome, data preprocessing, hyperparameters optimization, and performance measurement used. Sen, Sensitivity; Spe, Specificity; Accu, Accuracy; AUC, Area Under the Curve; PPV, Positive Predictive Value; NPV, Negative Predictive Value; NRI, Net Reclassification Index; ROC, Receiver Operating Curve; FPR, False Positive Rate; FNR, False Negative Rate, NA, Not Available

| **Author** | **Unbalanced outcome** | **A technique used for unbalance outcome** | **Data Preprocessing** | **Hyperparameters Optimization** | **Performance Measurement** |
| --- | --- | --- | --- | --- | --- |
| Almog et al. (2020) | Y | Oversampling | 70% training, 30% validation | NA | ROC, Sen, Spe, Precision, AUC |
| Bae et al. (2021) | Y | Data Augmentation | (1) Phase 1: Hospital A dataset is separated into training data (80%), internal validation data (10%), and test data (10%).  (2) Phase 2: Training, internal validation, and testing were conducted with all data from Hospitals A and B, using 80%, 10%, and 10% of each dataset, | NA | Sen, Spe, Accu, AUC, Other, Youden index |
| Beyaz et al. (2020) | Y | NA | NA | Genetic Algorithm | Sen, Spe, Accu, F1 score, Cohen’s kappa coefficient |
| Burns et al. (2017) | N | NA | NA | NA | Sensitivity, FPR, Free response ROC |
| Chen et al. (2021) | N | NA | 80% training 20% validation | NA | Sen, Spe, Accu, AUC |
| Chen et al. (2022) | Y | NA | 75% training, 25% validation | Trained with a batch size of 32 on each GPU. Optimized by the standard stochastic gradient descent algorithm. The learning rate decayed from 0.0125, and its warm-up epoch was 5. | Sen; Spe; Accu; AUC; ROC; 95% CI |
| Cheng et al. (2019) | Y | During the training process, image augmentation was applied with a zoom of 10%, horizontal flip, vertical flip, and rotation of 10 degree | 90% training, 10% validation,  PXR dataset was separated as 80% training and 20% validation | Batch size with 8, Adam optimizer, Initial learning rate was ${10}^{-3}$ with a reduced learning rate on the plateau, Final model was trained with 60 epochs under the optimal hyperparameters | Sen, Spe, Accu, AUC, FNR, FPR, F1 score, 95% CI, ROC |
| Cheng et al. (2020) | Y | NA | Randomly separated the development dataset into training (2163/3605, 60%), validation (721/3605, 20%), and testing (721/3605, 20%) | Adam optimizer with an initial learning rate of ${10}^{-3}$, Batch size was 8 and the DCNN was trained for 60 epochs without early stopping | Sen, Spe, Accu, AUC, FNR, FPR, F1 score, 95% CI, ROC, Cohen kappa |
| Cheng et al. (2021) | Y | NA | NA | NA | ROC, 95% CI of the AUC, Sen, Spe, Accu |
| Choi et al. (2020) | Y | Data augmentation by a factor of 5 using the Keras ImageDataGenerator, including horizontal flip, rotation, shift, shear, and zoom | 80% training 20% testing | Categorial cross-entropy as the loss function, stochastic gradient descent optimizer with a learning rate of $1 \times{10}^{-3}$ and a decay of $1 \times{10}^{-6}$, a momentum of 0.9 and Nesterov momentum, 31,625 iterations and a batch size of 4 | Sen, Spe, PPV, NPV, AUC, Precision-recall curve, ROC, 95% CI, McNemar test |
| Chou et al. (2022) | Y | NA | 60% training, 20% validation, 20% testing | NA | Sen, Spe, Accu, Kappa value |
| Chung et al. (2018) | Y | NA | Used 10-folds CV with 9-folds for training of model and 1-fold as testing | base_lr: 0.0001, max: 3, epochs, gamma: 0.1, weight_decay: 0.00001 | Sen, Spe, Accu, AUC, ROC, 95% CI, Youden Index |
| Derkatch et al. (2019) | Y | NA | 60% training, 10% validation, 30% testing | NA | Sen, Spe, Accu, AUC, ROC, 95% CI |
| Galassi et al. (2020) | Y | Synthetic Minority Over-sampling Technique | 70% training, 30% testing | Different kernels were considered in SVM: linear, linear with posterior probability regions, sigmoid, sigmoid with posterior probability regions, Gaussians, Gaussian with posterior probability regions, and Bayesian with posterior probability regions | Sen, Spe, Accu |
| Guermazi et al. (2022) | N | NA | 70% training, 10% validation, 20% testing | Stochastic gradient algorithm with a batch size of four. | Sen, Spe, ROC, 95% CI |
| Gupta et al. (2020) | N | Data Augmentation | 60% training, 20% validation, 20% testing | NA | Sen, Spe, PPV, NPV, Accu |
| Hayashi et al. (2022) | N | NA | 70% training, 10% validation, 20% testing | NA | Sen, Spe, ROC, 95% CI |
| Ho-Le et al. (2017) | Y | NA | 60% training, 40% testing | Cohen's Kappa coefficient as the metric for optimization | Sen, Spe, AUC |
| Inoue et al. (2022) | Y | NA | CNN was trained on 4174 axial slices, with 1043 axial slices held out for validation and 2447 axial slices for testing | Training parameters: a batch size of 1; a total of 200,000 iterations; and an initial learning rate of 0.0002 | Sen, F1 score, Precision |
| Kim et al. (2018) | N | Data Augmentation | 80% training, 10% validation, 10% testing | Learning rate and cycle number were optimized iteratively. The final model was trained using an initial learning rate of 0.02 and learning rate decay by factor 0.67 every 1,800 iterations | Sen, Spe, Accu, AUC, ROC |
| Kitamura et al. (2020) | N | NA | NA | NA | AUC, ROC |
| Korfiatis et al. (2018) | Y | Random Undersampling, Synthetic Minority Oversampling | 90% training, 10% testing | NA | Sen, Spe, G-Mean |
| Kruse et al. (2017) | Y | NA | 75% training, 25% testing | NA | Sen Spe, Accu, AUC, ROC |
| Lama et al. (2022) | Y | Data Augmentation | Cross-validation and test sets | Augmentation: translation, rotation, and mirroring | Sen, Spe, Accu, ROC, F1 score |
| Lemineur et al. (2007) | Y | NA | a different portion of train and testing data set: 50-50, 60-40, 80-20 | NA | Sen, Spe, Accu |
| Lindsey et al. (2018) | Y | NA | 90% training, 10% testing | Standard stochastic gradient descent algorithm (Adam), Early stopping | Sen, Spe, AUC, ROC, 95% CI |
| Liu et al. (2015) | Y | NA | 90% training, 10% testing | Different number of neurons in the hidden layers were utilized. The activation function of hidden neurons is a tangent sigmoid transfer function | Sen, Spe, Accu, PPV, NPV, AUC, ROC |
| Liu et al. (2022) | Y | NA | 700 JPG files were randomly divided into two datasets: a training database (including 643 files, consisting of 413 FIF and 230 normal hips, for AI learning and training) and the test dataset (including 57 files, consisting of 46 FIF and 11 normal hips, for effect validation). | Training database was first enhanced by the algorithm including image rollover, rotation, cropping, and blurring | Sen, Spe, Accu, AUC, ROC, F1 score, Precision, Average precision, Mean average precision, Missed diagnosis rate, Misdiagnosis rate, Time consumption |
| Mawatari et al. (2020) | Y | Data augmentation used with the allowance of plus and minus one degree, and horizontal flipping | NA (but used independent testing data) | NA | Sen Spe, Accu, AUC, ROC |
| Mehta et al. (2020) | Y | NA | 80% training, 20% testing | NA | Sen, Spe, Accu, AUC, ROC, 95% CI |
| Minonzio et al. (2020) | N | NA | 80% training, 20% testing | NA | Sen, Spe, ROC, AUC, Odds Ratio |
| Monchka et al. (2021) | Y | Oversampling, Data augmentation | 60% training, 10% validation, 30% testing | Adam stochastic gradient descent with a binary cross-entropy loss function, Cosine-based annealing learning rate scheduler (initial learning rate = ${10}^{-4}$) | Accu, Sen, Spe, PPV, NPV, balanced accuracy F1 score and AUC , Bootstrapping with 1000 samples was used to construct 95% Cis |
| Monchka et al. (2022) | Y | Active learning | 70% training, 30% testing | Adam stochastic gradient descent with a binary cross-entropy loss function, Cosine-based annealing learning rate scheduler (initial learning rate = ${10}^{-4}$) | Sen, Spe, AUC, F1 score, PPV, NPV, Balanced accuracy |
| Mu et al. (2021) | Y | NA | Training set (n = 610), tuning set (n = 100) which was used to select the final model. | Two augmentation methods: (i) flipping: flips input radiographs horizontally with a probability of 0.5; (ii) rotating | Sen, Spe, ROC, 95% CI |
| Murata et al. (2020) | N | NA | NA | Batch size and optimized epochs were assigned automatically by Visual Recognition V3 | Sen, Spe, Accu, 95% CI, ROC |
| Mutasa et al. (2020) | Y | Data augmentation: exposing the network to multiple small variations of each radiograph | 70% training, 20% validation, 10% testing | Learning rate set to ${10}^{-3}$, keep probability for dropout of 50%, moving average weight decay of 0.999, and L2 regularization weighting of ${10}^{-4}$. | Sen, Spe, Accu, PPV, NPV, AUC, ROC |
| Nguyen et al. (2022) | N | NA | 70% training, 10% validation, 20% testing | NA | Sen, Spe, ROC |
| Nishiyama et al. (2014) | Y | NA | NA | NA | AUC, Sen, Spe, Accu |
| Nissinen et al. (2021) | Y | NA | Used 10-folds CV with 9-folds for training of model and 1-fold as testing | Random search, Hyperband | Sen, Spe, Accu, AUC, ROC, 95% CI |
| Oakden-Rayner et al. (2022) | N | NA | NA | NA | Sen, Spe, AUC, ROC, 95% CI |
| Ozkaya et al. (2022) | N | NA | 50 images containing scaphoid fractures (6 displaced proximal pole, 6 non-displaced waists, 21 displaced waist, 6 non-displaced distal third, 4 displaced distal third and 7 occult scaphoid fractures) and 50 images containing healthy scaphoids were randomly picked and placed in the test set. | NA | Sen, Spe, AUC, F1 score, Youden index |
| Raghavendra et al. (2018) | Y | NA | 70% training, 30% testing | Learning rates: 0.1, 0.01, 0.001, 0.0001 | Accu, Sen, Spe |
| Raisuddin et al. (2021) | Y | NA | Three independent data sets were used for training and testing | Data augmentation: cutout32, jittering, random color padding on a particular side, downscaling, flipping, rotation, shearing, padding, salt and pepper, blur, noise, and gamma correction for the ROI localization block | Sen, Spe, Accu, AUC, F1 score, Kappa score, PPV |
| Ramos et al. (2022) | N | NA | 80% training, 20% testing | Grid search with 100 partitions | Sen, Spe, Accu, AUC, F1 score, Precision |
| Regnard et al. (2022) | Y | NA | 70% training, 10% validation 20% testing | NA | Sen, Spe, AUC, 95% CI, F1 score, PPV, NPV |
| Rosenberg et al. (2022) | Y | NA | Training set (N = 578) and a test set (N = 52), both containing a balanced mix | Augmentation: random rotation, flipping, and shifting. Used the Adam optimizer and learning rate by a factor of 0.1 if the accuracy did not improve for 10 epochs in a row | Sen, Spe, Accu |
| Salehinejad et al. (2021) | Y | NA | NA | Training setup of the BLSTM model was as follows: Adam optimizer with a learning rate of ${10}^{-6}$; 100 train-ing epochs; Batch size of 4; Input size of 2,048. | Sen, Spe, PPV, NPV, F1, Accu, MCC, AUC |
| Sato et al. (2021) | N | NA | 80% training 10% validation, 10% testing | NA | Sen, Spe, Accu, AUC, ROC, F-value |
| Small et al. (2021) | Y | NA | NA | NA | Sen, Spe, 95% CI, PPV, NPV, Kappa score |
| Su et al. (2019) | Y | NA | NA | Used a fixed complexity parameter (C_p = 0.01) to avoid the overfitting | Sen, Spe, AUC, NRI |
| Tomita et al. (2018) | N | NA | 80% training, 10% validation,  10% testing | NA | Accu, Sen,Spe |
| Tseng et al.(2013) | N | NA | 90% training, 10% testing | NA |  |
| Ulivier et al. (2021) | Y | NA | Used a re-sampling system called "TWIST" created by the Semeion Research Centre | NA | Sen, Spe, Accu, AUC |
| Urakawa et al. (2019) | Y | NA | 80% training, 10% validation,  10% testing | Exponential learning rate:  initial learning rate, 0.0001; decay steps, 265 iterations; and decay rate, 0.8. | Sen, Spe, Accu, AUC, 95% CI |
| Ureten et al. (2022) | N | Each image in the training raw dataset was flipped horizontally to increase the training data, and these images were again split into two to be used for training and validation (85% and 15%) | 75% training, 25% testing | Data augmentation: sharpness, brightness, contrast, and mirror symmetry | Sen, Spe, Accu, Precision |
| Wang et al. (2022) | Y | NA | NA | NA | Sen, Accu, AUC, F1 score, Precision |
| Wu et al. (2020) | Y | Oversampling | 80% training, 20% testing | NA | AUC; ROC; Accu |
| Yabu et al. (2021) | Y | NA | NA | NA | Sen, Spe, Accu, AUC, ROC, 95% CI |
| Yamada et al. (2020) | Y | NA | Paint 3D (Microsoft Corp, Redmond, WA, USA) by cropping the minimum region | 100 epochs with a learning rate of 0.1, Cross-entropy loss | Sen, Spe, Accu, F1 score |
| Yamamoto et al. (2020) | Y | Used only non-fracture data | 74 subjects training,  35 subjects testing | 100 epochs with a learning rate of 0.01, Stochastic gradient descent function was used as the optimization function for learning data | Sen, Spe, Accu, Prec |
| Yeh et al. (2022) | Y | Data Augmentation | NA | Cross-entropy, Adam optimizer, Learning rate was set to 0.0001, Parameters were initialized using ImageNet, Batch size was set to 32, Epochs was set to 100,  L2 regularization, Early stopping | Sen, Spe, Accu, Other: McNemar test |
| Yi-Chu Li et al. (2021) | Y | Undersampling of normal vertebrae was performed for the training and validation datasets | 60% training, 20% validation, 20% testing | NA | Sen, Spe, Accu, Kappa, ROC, AUC |
| Yoda et al. (2022) | Y | Data Augmentation | NA | 100 epochs with a learning rate of 0.1, Cross-entropy loss. | Sen, Spe, Accu, AUC, ROC |
| Yoon et al. (2021) | Y | Data Augmentation | 70% training, 10% validation, 20% testing | NA | Sen, Spe, AUC, ROC, 95% CI, PPV, NPV |
| Yu et al. (2020) | N | NA | 60% training, 20% validation, 20% testing | Cross-entropy loss function using a stochastic gradient descent optimizer | AUC, Sen, Spe |
| Yuan Li et al. (2021) | Y | NA | Used 10-folds CV with 9-folds for training of model and 1-fold as testing | NA | Sen, Spe, Accu |
