# Supplementary material for: Artificial intelligence in fracture detection with different image modalities and data types: A systematic review and meta-analysis
Source: PLOS Digit Health. 2024 Jan 30;3(1):e0000438. doi: 10.1371/journal.pdig.0000438 (PMC10826962; doi:10.1371/journal.pdig.0000438)
Supplement: S4 Table — (DOCX) [file pdig.0000438.s006.docx]

**S4 Table**. A summary of the contingency table for 66 selected studies. TP, True Positive; FP, False Positive; FN, False Negative; TN, True Negative; AUC, Area Under the Curve; NA, Not Available

| **Author** | **TP** | **FP** | **FN** | **TN** | **Accuracy** | **AUC** |
| --- | --- | --- | --- | --- | --- | --- |
| Almog et al. (2020) | 10277 | 19235 | 1967 | 37719 | 0.69 | 0.81 |
| Bae et al. (2021) | 1071 | 22 | 38 | 3058 | 0.99 | NA |
| Beyaz et al. (2020) | 1111 | 207 | 230 | 558 | 0.79 | NA |
| Burns et al. (2017) | 201 | 9 | 48 | 162 | 0.86 | NA |
| Chen et al. (2021) | 65 | 47 | 25 | 126 | 0.73 | 0.72 |
| Chen et al. (2022) | 422 | 140 | 106 | 298 | 0.75 | NA |
| Cheng et al. (2019) | 49 | 8 | 1 | 42 | 0.91 | 0.98 |
| Cheng et al. (2020) | 49 | 8 | 1 | 42 | 0.91 | 0.91 |
| Cheng et al. (2021) | 381 | 15 | 0 | 1492 | 0.99 | 0.97 |
| Choi et al. (2020) | 23 | 10 | 0 | 62 | 0.89 | 0.98 |
| Chou et al. (2022) | 302 | 103 | 37 | 1622 | 0.93 | NA |
| Chung et al. (2018) | 1362 | 14 | 15 | 500 | 0.98 | 1 |
| Derkatch et al. (2019) | 534 | 373 | 77 | 2838 | 0.88 | 0.94 |
| Galassi et al.(2020) | 74 | 4 | 15 | 44 | 0.86 | NA |
| Guermazi et al. (2022) | 4331 | 256 | 1429 | 5504 | 0.85 | NA |
| Gupta et al. (2020) | 300 | 7 | 7 | 140 | 0.97 | 0.99 |
| Hayashi et al. (2022) | 137 | 15 | 13 | 135 | 0.91 | NA |
| Ho-Le et al. (2017) | 75 | 132 | 15 | 945 | 0.87 | 0.85 |
| Inoue et al. (2022) | 288 | 156 | 78 | 1925 | 0.90 | NA |
| Kim et al. (2018) | 626 | 70 | 83 | 611 | 0.89 | 0.95 |
| Kitamura et al. (2020) | 3362 | 547 | 206 | 3222 | 0.90 | 0.95 |
| Korfiatis et al. (2018) | 279 | 9457 | 34 | 53933 | 0.85 | 0.92 |
| Kruse et al. (2017) | 299 | 969 | 41 | 4130 | 0.81 | 0.92 |
| Lama et al. (2022) | 10 | 0 | 1 | 9 | 0.95 | NA |
| Lemineur et al. (2007) | 13 | 5 | 6 | 37 | 0.82 | NA |
| Lindsey et al. (2018) | 239 | 27 | 73 | 1061 | 0.93 | 0.99 |
| Liu et al. (2015) | 192 | 75 | 36 | 422 | 0.85 | 0.91 |
| Liu et al. (2022) | 40 | 1 | 6 | 10 | 0.88 | NA |
| Mawatari et al. (2020) | 22 | 7 | 3 | 18 | 0.80 | 0.91 |
| Mehta et al. (2020) | 18 | 1 | 4 | 38 | 0.92 | 0.90 |
| Minonzio et al. (2020) | 106 | 7 | 15 | 122 | 0.91 | NA |
| Monchka et al. (2021) | 556 | 402 | 79 | 2785 | 0.87 | 0.94 |
| Monchka et al. (2022) | 580 | 454 | 66 | 2722 | 0.86 | NA |
| Mu et al. (2021) | 106 | 18 | 9 | 56 | 0.86 | NA |
| Murata et al. (2020) | 127 | 23 | 19 | 131 | 0.86 | NA |
| Mutasa et al. (2020) | 671 | 66 | 23 | 303 | 0.92 | 0.87 |
| Nguyen et al. (2022) | 125 | 15 | 25 | 135 | 0.87 | NA |
| Nishiyama et al. (2014) | 33 | 2 | 4 | 31 | 0.91 | 0.94 |
| Nissinen et al. (2021) | 255 | 1035 | 170 | 1489 | 0.59 | 0.63 |
| Oakden-Rayner et al. (2022) | 38 | 1 | 2 | 40 | 0.96 | NA |
| Ozkaya et al. (2022) | 38 | 4 | 12 | 46 | 0.84 | NA |
| Raghavendra et al. (2018) | 692 | 31 | 8 | 389 | 0.97 | NA |
| Raisuddin et al. (2021) | 125 | 10 | 4 | 68 | 0.93 | NA |
| Ramos et al. (2022) | 199 | 41 | 41 | 199 | 0.83 | NA |
| Regnard et al. (2022) | 768 | 479 | 17 | 3517 | 0.90 | NA |
| Rosenberg et al. (2022) | 21 | 3 | 3 | 25 | 0.88 | NA |
| Salehinejad et al. (2021) | 67 | 37 | 22 | 82 | 0.72 | 0.71 |
| Sato et al. (2021) | 159 | 4 | 8 | 129 | 0.96 | 0.99 |
| Small et al. (2021) | 109 | 17 | 34 | 505 | 0.92 | NA |
| Su et al. (2019) | 112 | 1069 | 60 | 4558 | 0.81 | 0.73 |
| Tomita et al. (2018) | 69 | 2 | 12 | 46 | 0.89 | NA |
| Tseng et al. (2013) | 170 | 54 | 47 | 163 | 0.77 | 0.87 |
| Ulivier et al. (2021) | 70 | 23 | 13 | 66 | 0.79 | 0.83 |
| Urakawa et al. (2019) | 1665 | 108 | 41 | 1532 | 0.96 | NA |
| Ureten et al. (2022) | 66 | 6 | 2 | 61 | 0.94 | NA |
| Wang et al. (2022) | 119 | 59 | 23 | 370 | 0.86 | NA |
| Wu et al. (2020) | 221 | 45 | 48 | 712 | 0.91 | 0.71 |
| Yabu et al. (2021) | 88 | 22 | 12 | 160 | 0.88 | 0.95 |
| Yamada et al. (2020) | 200 | 2 | 0 | 98 | 0.99 | NA |
| Yamamoto et al. (2020) | 25991 | 17308 | 17009 | 51892 | 0.69 | NA |
| Yeh et al. (2022) | 47 | 12 | 3 | 128 | 0.92 | NA |
| Yi-Chu Li et al. (2021) | 129 | 45 | 12 | 644 | 0.93 | 0.99 |
| Yoda et al. (2022) | 49 | 2 | 1 | 45 | 0.97 | NA |
| Yoon et al. (2021) | 900 | 469 | 25 | 910 | 0.79 | NA |
| Yu et al. (2020) | 297 | 9 | 10 | 300 | 0.97 | 0.99 |
| Yuan Li et al. (2021) | 281 | 27 | 15 | 110 | 0.90 | NA |
