# Supplementary material for: Artificial intelligence in fracture detection with different image modalities and data types: A systematic review and meta-analysis
Source: PLOS Digit Health. 2024 Jan 30;3(1):e0000438. doi: 10.1371/journal.pdig.0000438 (PMC10826962; doi:10.1371/journal.pdig.0000438)
Supplement: S5 Table — TF: Trim and Fill method, DOR: Diagnostic Odds Ratio, CI: Confidence Interval. (DOCX) [file pdig.0000438.s007.docx]

**S5 Table**. Summary of Publication Bias Assessment across different fracture outcomes. TF: Trim and Fill method, DOR: Diagnostic Odds Ratio, CI: Confidence Interval.

| **Outcome** | **No. of Studies included** | **No. of Studies after TF** | **DOR (95% CI)** | **DOR after TF (95% CI)** |
| --- | --- | --- | --- | --- |
| Hip | 18 | 28 | 99.5 (39.4-251.5) | 7.1 (1.5-33.5) |
| Vertebral | 20 | 28 | 38.3 (21.4-68.5) | 17.5 (8.9-34.1) |
| Wrist | 3 | 5 | 105.7 (56.4-197.9) | 65.7 (31.4-137.6) |
| Femoral Neck | 4 | 6 | 125.8 (10.9-1444.7) | 16.1 (2.5-87.4) |
| Multiple* | 11 | 17 | 88.7 (33.5-234.6) | 16.9 (3.6-78.7) |
| Others** | 10 | 14 | 179.7 (65.9-489.5) | 63.4 (19.3-208.4) |

* Multiple fractures outcome studies include hip and pelvic (2), hip and spine (1), major osteoporotic fractures (1), multiple (3), osteoporotic fractures (2), pelvic and limbs (1), pelvic, spine, and rib (1).

** Others fracture outcome studies include scaphoid (2), thoracolumbar (2), cervical spine (1), hand (1), lumber spine (1), proximal humerus (1), supracondylar (1), and trabecular bone (1).
