# Supplementary material for: Artificial intelligence in fracture detection with different image modalities and data types: A systematic review and meta-analysis
Source: PLOS Digit Health. 2024 Jan 30;3(1):e0000438. doi: 10.1371/journal.pdig.0000438 (PMC10826962; doi:10.1371/journal.pdig.0000438)
Supplement: S6 Table — TF: Trim and Fill method, DOR: Diagnostic Odds Ratio, CI: Confidence Interval. (DOCX) [file pdig.0000438.s008.docx]

**S6 Table**. Summary of Publication Bias Assessment across different data types. TF: Trim and Fill method, DOR: Diagnostic Odds Ratio, CI: Confidence Interval.

| **Outcome** | **No. of Studies included** | **No. of Studies after TF** | **DOR (95% CI)** | **DOR after TF (95% CI)** |
| --- | --- | --- | --- | --- |
| Tabular | 9 | 14 | 20.1 (12.1-33.2) | 10.4 (5.7-18.9) |
| Image | 54 | 81 | 104.2 (65.1-166.7) | 10.2 (4.4-23.6) |
| Tabular and Image | 3 | 5 | 73.2 (27.2-196.5) | 54.3 (22.7-130.1) |
