# Supplementary material for: Artificial intelligence in fracture detection with different image modalities and data types: A systematic review and meta-analysis
Source: PLOS Digit Health. 2024 Jan 30;3(1):e0000438. doi: 10.1371/journal.pdig.0000438 (PMC10826962; doi:10.1371/journal.pdig.0000438)
Supplement: S7 Table — (DOCX) [file pdig.0000438.s009.docx]

**S7 Table**. Overview of Key Challenges and Potential Resolutions in the Utilization of Machine Learning or Deep Learning for Fracture Diagnosis.

| **Major Issues** | **Potential Solutions** |
| --- | --- |
| Limited Dataset Size | Augment data using data synthesis or generative adversarial networks (GANs). Collaborate with multiple institutions for data sharing. |
| Class Imbalance | Utilize techniques such as oversampling, undersampling, or different loss functions to address class imbalance. |
| Feature Engineering | Utilize domain knowledge to extract relevant features and employ automatic feature selection methods. |
| Model Overfitting | Implement techniques like dropout, regularization, and early stopping. Use cross-validation for robustness assessment. |
| Interpretability | Employ explainable AI methods (e.g., SHAP values, LIME) to enhance model interpretability and transparency. |
| Generalization | Apply transfer learning, fine-tuning pre-trained models to adapt them to specific fracture diagnosis tasks. |
| Lack of Standardization | Establish standardized protocols for data collection, preprocessing, and model evaluation. |
| Limited Real-world Data | Collaborate with medical professionals to obtain diverse and representative real-world data. |
| Hardware and Computation | Optimize model architectures and utilize cloud computing resources to handle large datasets and complex models. |
| Ethical Considerations | Address data privacy concerns and ensure compliance with ethical guidelines and regulations. |
