# Supplementary material for: Artificial intelligence in fracture detection with different image modalities and data types: A systematic review and meta-analysis
Source: PLOS Digit Health. 2024 Jan 30;3(1):e0000438. doi: 10.1371/journal.pdig.0000438 (PMC10826962; doi:10.1371/journal.pdig.0000438)
Supplement: S1 Fig — (DOCX) [file pdig.0000438.s010.docx]

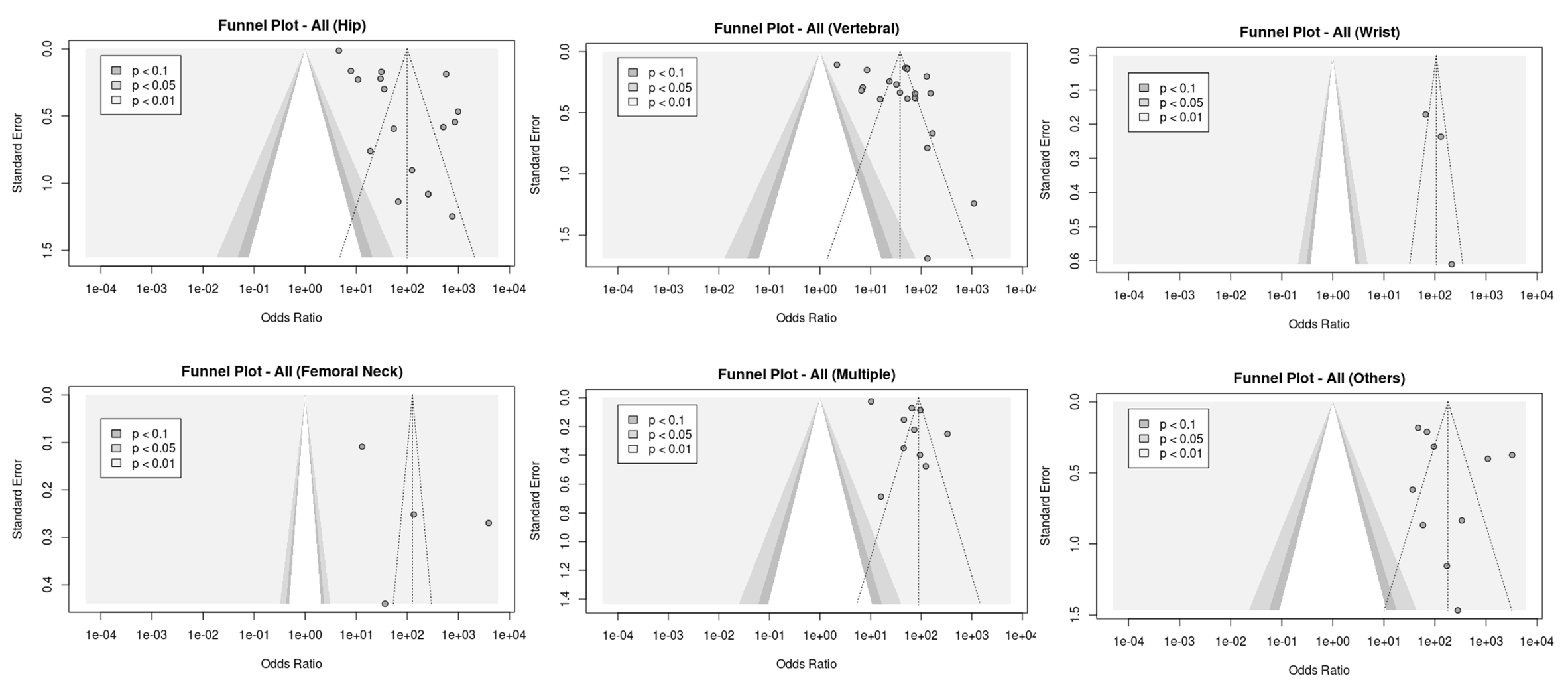
 **S1 Fig.** Contour-Enhanced Funnel Plot for Publication Bias Assessment across Different Fracture Outcomes.
