# Supplementary material for: Artificial intelligence in fracture detection with different image modalities and data types: A systematic review and meta-analysis
Source: PLOS Digit Health. 2024 Jan 30;3(1):e0000438. doi: 10.1371/journal.pdig.0000438 (PMC10826962; doi:10.1371/journal.pdig.0000438)
Supplement: S2 Fig — The open circle represents the “filled” studies from the Trim & Fill Method in each fracture outcome plot. (DOCX) [file pdig.0000438.s011.docx]

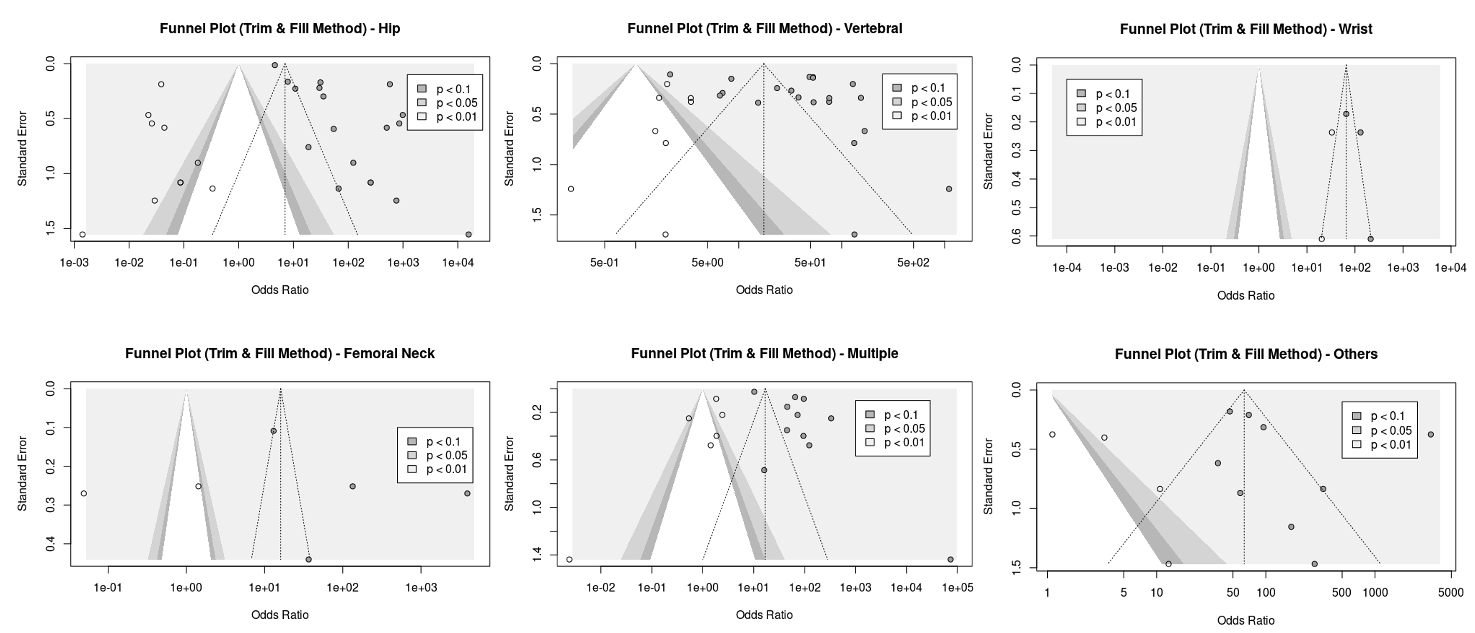
**S2 Fig**. Contour-Enhanced Funnel Plot for Publication Bias Assessment across Different Fracture Outcomes after employing the Trim & Fill Method. In each fracture outcome plot, the open circle represents the “filled” studies from the Trim & Fill Method.
