# Supplementary material for: Artificial intelligence in fracture detection with different image modalities and data types: A systematic review and meta-analysis
Source: PLOS Digit Health. 2024 Jan 30;3(1):e0000438. doi: 10.1371/journal.pdig.0000438 (PMC10826962; doi:10.1371/journal.pdig.0000438)
Supplement: S3 Fig — The top row illustrates the funnel plot encompassing all studies. The second row shows the Contour-Enhanced Funnel Plot for Publication Bias Assessment after employing the Trim & Fill Method. The open circle designates the studies “filled” through the Trim & Fill Method within each contour-enhanced funnel plot in the second row. (DOCX) [file pdig.0000438.s012.docx]

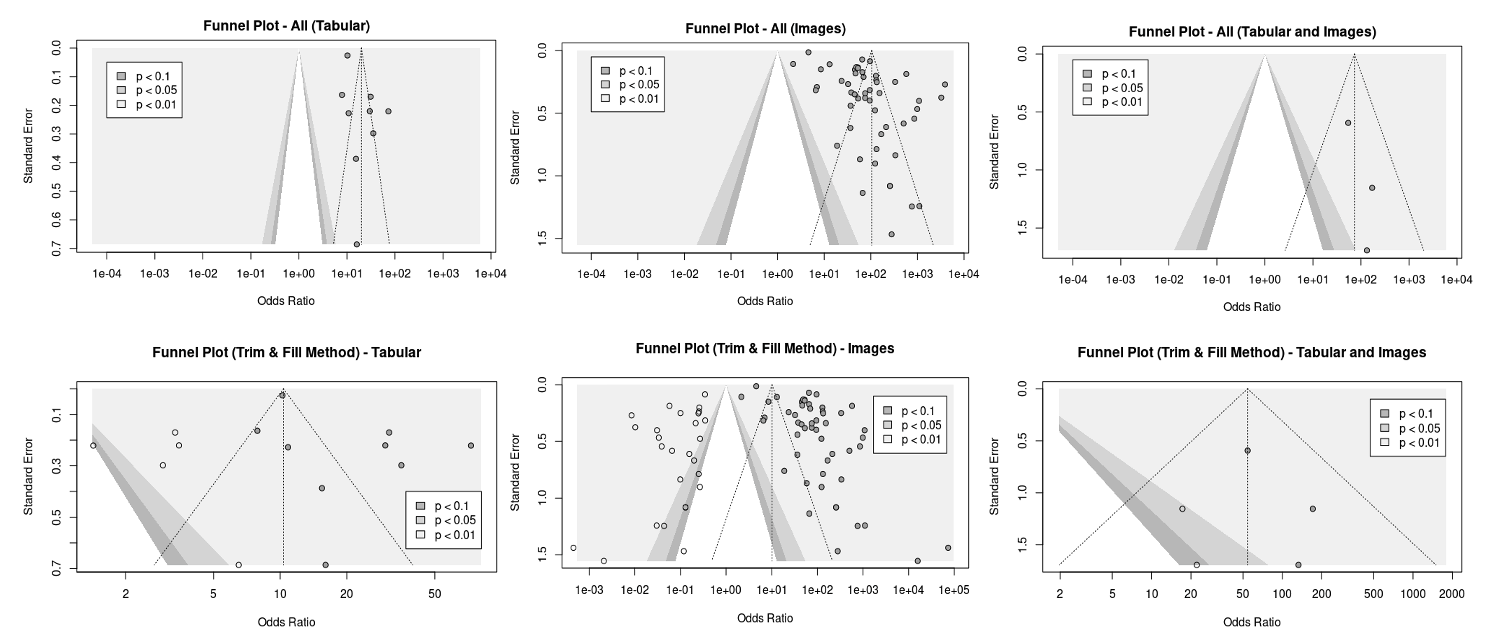
**S3 Fig.** Contour-Enhanced Funnel Plot: Evaluating Publication Bias Across Various Data Types. The top row illustrates the funnel plot encompassing all studies. The second row shows the Contour-Enhanced Funnel Plot for Publication Bias Assessment after employing the Trim & Fill Method. Within each contour-enhanced funnel plot in the second row, the open circle designates the studies "filled" through the Trim & Fill Method.
